# Site-Specific Glycosylation Profiling of Protein Subunit and Inactivated Virus Vaccines

**DOI:** 10.3390/vaccines14070644

**Published:** 2026-07-22

**Authors:** Zachary C. Goecker, Meghan C. Burke, Yi Liu, Yuri A. Mirokhin, Sergey L. Sheetlin, Guanghui Wang, Dmitrii V. Tchekhovskoi, Xiaoyu Yang, Stephen E. Stein

**Affiliations:** Mass Spectrometry Data Center, National Institute of Standards and Technology, 100 Bureau Drive, Gaithersburg, MD 20899, USA

**Keywords:** vaccine comparability, subunit vaccine, inactivated vaccine, glycosylation, site-specific, mass spectrometry, proteomics

## Abstract

**Background/Objectives**: Glycosylation can affect vaccine antigen structure and function, making site-specific glycan characterization relevant to antigen quality and comparability. However, quantitative approaches for comparing glycan microheterogeneity remain limited. This study evaluated the utility of the glycopeptide abundance distribution spectra framework for measuring similarity among site-specific glycosylation profiles in vaccines and antigen reference reagents across manufacturing conditions. **Methods**: Intact N-linked glycopeptides were characterized by nanoflow liquid chromatography–tandem mass spectrometry with stepped-energy fragmentation. Products included monovalent and quadrivalent influenza antigens produced in embryonated eggs, Madin–Darby canine kidney cells, or *Spodoptera frugiperda* cells, together with a SARS-CoV-2 spike vaccine produced in *Spodoptera frugiperda* cells and a Chinese hamster ovary cell-produced varicella-zoster virus glycoprotein E vaccine. Site-specific glycan distributions were represented as distribution spectra and compared using NIST MS Search software. Dot-product scores ranging from 0 to 999 quantified similarity. **Results**: Across measured glycosylation sites, distributions clustered into six recurrent classes. Similarity was high for replicate analyses, conserved influenza components across annual formulations, and matched components from different suppliers within the same production platform (similarity scores = 978, 961, and 960, respectively). Similarity was lower between sites within the same protein, between influenza strains, and between production sources (similarity scores = 554, 540, and 209, respectively). Among production-source comparisons, egg- and Madin–Darby canine kidney-derived profiles were most similar, and the overall ordering of glycosylation similarity was consistent with broad phylogenetic relatedness among production hosts. **Conclusions**: Distribution spectra-based similarity scoring of vaccine glycoproteins provides a quantitative, reusable approach for documenting site-specific glycosylation microheterogeneity. Using this method, we can conclude that production source is the dominant contributor to variation, whereas replicates, annual formulations, and suppliers within the same production platform are highly consistent.

## 1. Introduction

Site-specific glycosylation analysis is a mass spectrometry-based measurement that fragments intact glycopeptides to determine the identities and relative abundances of attached glycans for each glycosylation site on a protein [1]. In contrast, released-glycan profiling (i.e., glycomics) and intact-glycoprotein analyses typically provide glycan or glycoform information aggregated across all sites on a protein and therefore do not directly resolve site-to-site differences in occupancy and pattern (i.e., microheterogeneity) [2]. By resolving glycosylation at the site level, intact glycopeptide measurements enable reproducible comparison of glycosylation distributions across samples and biomanufacturing conditions, supporting both production comparability and mechanistic interpretation of glycoprotein function [3,4,5,6].

Reproducibility in biomanufacturing of biologics and vaccines is central to quality control and product comparability. While regulatory guidelines do not prescribe a single analytical method for evaluating glycosylation microheterogeneity, they do emphasize defining patterns of heterogeneity and demonstrating consistency between production lots as part of an overall control strategy. The International Council for Harmonisation (ICH) guideline Q6B on test procedures and acceptance criteria for biotechnological/biological products describes the need to “define the pattern of heterogeneity” of the product and establish acceptance criteria that can be used to demonstrate manufacturing consistency [7]. Likewise, within the United States, the Food and Drug Administration 21 CFR Part 211 on current good manufacturing practice for finished pharmaceuticals describes the need for suitable testing to “assure that batches of drug products meet each appropriate specification” to demonstrate consistency [8]. Within this framework, quantitative assessment of similarity between site-specific glycosylation profiles can provide an orthogonal, information-rich readout of product heterogeneity that may be useful for comparability assessments and investigations of process-related changes.

The extent to which glycosylation microheterogeneity influences biological activity is increasingly being quantified across vaccines and therapeutics. In multiple systems, glycan composition and processing state can modulate antigenicity, receptor interactions, and immune recognition. For influenza virus hemagglutinin (HA), differences in glycan processing have been associated with altered antibody responses in animal models, including comparisons of high-mannose-, complex-type-, and minimally processed glycan presentations [9,10]. For SARS-CoV-2 spike subunit antigens, complex-type glycans have been associated with stronger neutralizing immune responses relative to high-mannose-enriched forms in experimental systems [5]. For erythropoietin (EPO), sialylated N-glycans are linked to enhanced in vivo biological activity, reflecting well-established relationships between glycosylation and pharmacology [6]. Collectively, these studies motivate analytical strategies that can resolve and compare site-specific glycosylation patterns when characterizing vaccine antigens and therapeutic glycoproteins.

Influenza vaccines are particularly well suited for site-specific glycosylation analysis because they contain two major glycoprotein antigens, HA and neuraminidase (NA), and are produced using multiple manufacturing platforms (e.g., egg-based, mammalian cell-based, and recombinant expression systems) that impose distinct host-specific glycan-processing constraints. HA is the primary target of many influenza vaccine responses because it mediates viral attachment and entry by binding sialylated host-cell receptors, making it a determinant of host and cell tropism as well as a major antigenic target of the humoral immune response. Antibodies that block HA-mediated receptor binding are commonly assessed using hemagglutination inhibition assays, which provide a functional measure of antigenic similarity and help guide selection of candidate influenza vaccine viruses. The HA and NA genes of circulating influenza viruses undergo antigenic drift, which can alter primary sequences, introduce or remove glycosylation sequons, and thereby affect glycan occupancy and processing at specific sites. This work focuses on commercially available influenza protein subunit and inactivated virus vaccines because they are widely administered and span multiple production sources, including egg-based preparations that can exhibit egg-adaptive mutations as well as more recent alternatives such as MDCK cell-derived and Sf9 recombinant vaccines. Although HA has historically been emphasized in influenza vaccine characterization in both a research and quality control context because of its central role in receptor binding, antigenicity, and HAI-based vaccine-strain selection, NA also contributes to protective immunity and elicits measurable vaccine responses in a substantial fraction of recipients [11]. As NA-containing vaccines continue to evolve, analytical characterization of NA, alongside HA, may become increasingly relevant. To assess whether the same analytical framework extends beyond influenza vaccines, we also included a SARS-CoV-2 protein subunit vaccine and a shingles vaccine. These products broaden the study to structurally distinct viral glycoproteins with different numbers of N-linked glycosylation sites and different production hosts, namely Sf9 insect cells and CHO cells. Their inclusion therefore expands the range of antigen and manufacturing contexts represented in the dataset while keeping influenza vaccines as the primary focus of the study.

**Figure 1:**
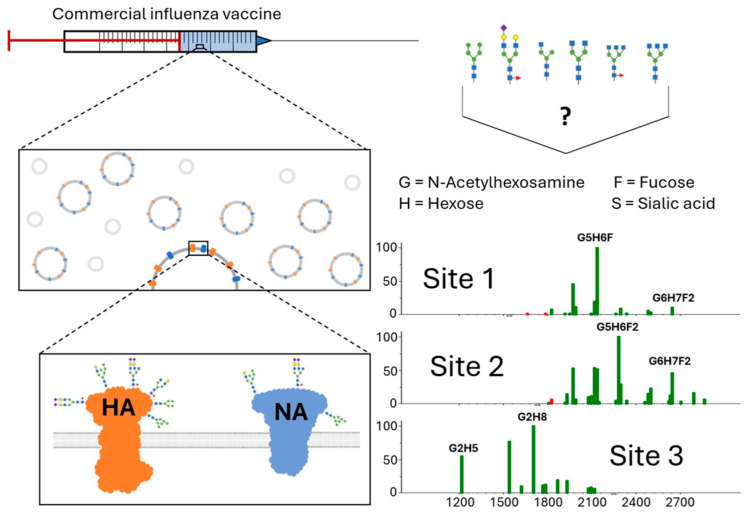
Illustration of site-specific glycosylation analysis of vaccines. This depiction summarizes the site-specific glycosylation methods used in this study to measure glycosylation patterns of viral glycoproteins, including hemagglutinin (HA) and neuraminidase (NA). Commercial vaccines and antigen reference reagents were processed, and glycopeptide abundance distribution spectra (GADS) were assigned to each N-linked glycosylation site to assess variability.

Here, we use nanoflow liquid chromatography coupled with stepped-energy fragmentation and high-resolution Orbitrap mass spectrometry to identify intact N-linked glycopeptides from monovalent influenza antigen reference reagents, quadrivalent influenza vaccines, a recombinant varicella-zoster vaccine, and a SARS-CoV-2 protein subunit vaccine (Figure 1). We focus on N-linked glycosylation because robust workflows for site-specific N-glycopeptide analysis are established; in contrast, site-specific O-glycosylation measurements are less established and often require alternative proteases and specialized workflows [12]. To quantitatively compare site-specific glycosylation distributions across replicate preparations, production years, suppliers, influenza vaccine virus components, and production sources, we represent each site-specific distribution as a glycopeptide abundance distribution spectrum (GADS) and compute similarity using dot-product scoring from tools developed by the NIST Mass Spectrometry Data Center [13]. We hypothesized that GADS-based dot-product scoring would reproducibly quantify site-specific glycosylation similarity across complex vaccine products and distinguish analytical reproducibility from variation associated with biological and manufacturing differences.

## 2. Materials and Methods

### 2.1. Vaccines and Glycoproteins

In total, 15 vaccines and antigen reagents were obtained for site-specific glycosylation analysis. See Table 1 for a complete list of products and Table S1 for a list of corresponding suppliers. Six reagents are from single-strains of influenza A, seven are quadrivalent influenza vaccines, one is a SARS-CoV-2 vaccine (supplier 5, 2023–2024 formulation), and one is a varicella-zoster vaccine (supplier 6). From supplier 1, national drug codes of relevant products include NDC 33332-421-10, NDC 33332-422-11, NDC 33332-423-10, NDC 70461-123-03, and NDC 70461-423-10. For supplier 2, product numbers of relevant products include 14/254, 83/537, and 01/614. For supplier 3, catalog numbers of relevant products include H1N12099-209I, H3N20799-215I, and H3N2993-216I. For supplier 4, the national drug code of the relevant product is NDC 49281-0722-88. For supplier 5, the national drug code of the relevant product is NDC 80631-105-02. For supplier 6, the national drug code of the relevant product is NDC 58160-823-11. For the influenza vaccines, glycoproteins hemagglutinin (HA) and neuraminidase (NA) are targeted for site-specific glycosylation analysis. For the COVID-19 vaccine, the spike (S) protein is targeted. For the varicella-zoster vaccine, surface glycoprotein E (gE) is the target. These vaccines and reagents were selected based on their use in relevant literature [14,15,16,17], availability among multiple suppliers, diversity of expression systems used, and broad range of glycosylation sites. Note that the vaccine with the largest annual administration is the influenza vaccine, with about 150 million doses distributed annually in the United States [18]. Therefore, our main focus and analysis is on commercially available influenza vaccines.

### 2.2. Protein Digestion and Desalting

Protein digestion and sample cleanup were performed using procedures derived from earlier work [3]. Samples were processed by either an in-solution or an in-gel workflow. The in-solution workflow was used for vaccines without adjuvants or amphipathic additives such as Tween-20 or Triton X-100, including AFLQ-21, AFLQ-22, AFLQ-23, FCVX-23, the supplier 2 monovalent vaccines (NIBSC-NC99, NIBSC-PH82, and NIBSC-SW13), the supplier 3 monovalent vaccines (CB-PA99, CB-NC99, and CB-SD93), and Shingrix. This procedure was adapted from Cyr et al. [19]. For each sample, 10 µg of estimated protein was adjusted to a final volume of 1 mL with 50 mmol/L ammonium bicarbonate (ABC) (Thermo Fisher Scientific, Waltham, MA, USA). The sample was transferred to a Spectra/Por7 dialysis membrane with a 1 kDa molecular mass cutoff (Avantor, Radnor, PA, USA) and dialyzed against 10 mmol/L ABC in a 1 L reagent bottle maintained in a stirred ice bath. Two successive dialysis periods of 2 h were followed by an overnight dialysis. The dialyzed sample was concentrated to dryness in Aqueous mode at 40 °C for 1 h, or until dry, using a Genevac EZ-2 Plus SpeedVac (SP Scientific, Warminster, PA, USA), and the residue was reconstituted in 50 µL of water purified with a Barnstead Pacific RO Milli-Q system (Thermo Fisher Scientific). Dithiothreitol (DTT) (Sigma-Aldrich, St. Louis, MO, USA) was added to 20 mmol/L, and reduction was carried out for 1 h at 60 °C and 450 rpm in an Eppendorf ThermoMixer C (Eppendorf, Hamburg, Germany). After the sample cooled to room temperature, iodoacetamide (IAA) (Sigma-Aldrich) was added to 55 mmol/L. Alkylation then proceeded for 45 min at room temperature and 450 rpm in the ThermoMixer C with the tube cap covered. Following alkylation, the sample was again dialyzed, dried, and reconstituted using the procedure described above.

Five proteases from two suppliers were used for vaccine-protein digestion: sequencing-grade trypsin (Promega V511A, Madison, WI, USA), Glu-C (Promega V165A), Lys-C (Promega V167A), chymotrypsin (Promega V106A), and alpha-lytic WT protease (Sigma-Aldrich A6362). The six digestion conditions comprised trypsin/Glu-C, trypsin/Lys-C, trypsin/chymotrypsin, chymotrypsin/Glu-C, chymotrypsin alone, and alpha-lytic WT protease alone. For the trypsin/Glu-C and trypsin/Lys-C digestions, both enzymes were added simultaneously at an enzyme-to-substrate mass ratio of 1:50 for each enzyme, and samples were mixed for 18 h at 37 °C and 450 rpm in a ThermoMixer C. The chymotrypsin, trypsin/chymotrypsin, and Glu-C/chymotrypsin conditions also used simultaneous enzyme addition at a 1:50 enzyme-to-substrate mass ratio for each enzyme, but incubation was performed for 18 h at room temperature and 450 rpm. Alpha-lytic protease was added at an enzyme-to-substrate mass ratio of 1:100 and incubated for 1 h at 37 °C and 450 rpm; a second addition was then made and incubated under the same conditions. Digestion was terminated by adding HPLC-grade trifluoroacetic acid (TFA) (Fisher Chemical, Fair Lawn, NJ, USA) to a final volume fraction of 0.2%.

The second method of digestion used in-gel techniques adapted from previous work [20]. This method of protein digestion was performed on vaccines which contained adjuvants or strong surfactants. This includes vaccines FCVX-22, FBLK-22, FLAD-23, and NVX-23 from supplier 5. Buffer exchange and protein gel electrophoresis were conducted as follows. A total of 20 µg of estimated protein was buffer exchanged for each sample using a 0.5 mL, 3 kDa molecular mass cutoff Amicon centrifugal filter (MilliporeSigma, Burlington, MA, USA) and spun at 14,000× *g* for 30 min in a Microfuge 22R Centrifuge (Beckman Coulter, Brea, CA, USA). A total of 400 µL of 25 mmol/L ABC was added to the filter and the device was again spun at 14,000× *g* for 30 min. The filter was then inverted into a new tube and spun at 1000× *g* for 2 min. A total of 100 µL of 25 mmol/L ABC was added to the filter membrane and repeatedly pipetted in and out for agitation, with care as to not touch the membrane. The filter was again inverted into the tube and spun at 1000× *g* for 2 min. This process was repeated twice more for a total of approximately 300 µL of protein concentrate. After buffer exchange, each sample was dried down using the Genevac EZ-2 plus Speedvac in Aqueous mode at 40 °C for about 1 h or until dry. Each sample was then reconstituted in 13 µL of Milli-Q water. The protocol was then followed from NuPAGE Bis-Tris mini gels (ThermoFisher) [21]. Briefly, 5 µL of LDS buffer (4×) (Invitrogen, Carlsbad, CA, USA) and 2 µL of DTT (10×) were added to the sample and heated at 70 °C for 10 min. Samples were loaded onto a NuPAGE (4 to 12) % Bis-Tris gel (1.5 mm-thick, 10 well gel) (Invitrogen), and were run using 1× MES buffer (Invitrogen) at 200 V constant for 5 min using a PowerPac Basic power supply (Bio-Rad, Hercules, CA, USA) coupled to a Novex Mini-Cell (Invitrogen). The gel was then rinsed with Milli-Q water for 5 min with gentle shaking. The rinse was repeated two more times. 20 mL of SimplyBlue SafeStain (Invitrogen) was added to the gel and the gel was gently shaken for 1 h. The staining solution was then discarded and Milli-Q water was added to rinse the gel for 1 h. The water rinse was repeated once more for 1 h. The stained section of each lane was then excised using a sterilized razor blade and cut into pieces approximately 1 mm × 1 mm.

In-gel digestion of vaccine proteins started with gel destaining. Gel pieces were destained with 150 µL of 25 mmol/L ABC containing a 50% volume fraction of acetonitrile (ACN) (Supelco, Bellefonte, PA, USA) for 10 min. Destaining was performed two more times. Gel pieces were then dried down in the speedvac for 30 min or until gel pieces were white. Gel pieces were then rehydrated with 150 µL of 20 mmol/L DTT and incubated at 60 °C for 1 h at 450 rpm. After reduction, excess liquid was removed from the gel pieces and 150 µL of 55 mmol/L IAA was added to each sample. Alkylation proceeded with incubation at room temperature for 45 min at 450 rpm in the dark. After alkylation, excess liquid was removed from the gel pieces and 150 µL of 25 mmol/L ABC was added to the tube to rinse the gel pieces for 10 min. Supernatant was removed and the gel pieces were then dehydrated with 150 µL of 25 mmol/L ABC containing a 50% volume fraction of ACN and let sit for 10 min. The supernatant was removed and the dehydration was repeated two more times. The samples were then dried down using a speedvac for 30 min or until gel pieces were white. Gel pieces were then rehydrated on ice using 150 µL of different chilled protease solutions. Protease combinations were as previously described for the in-solution digestion. For trypsin/Glu-C, trypsin/Lys-C, trypsin/chymotrypsin, chymotrypsin, and Glu-C/chymotrypsin, all proteases were at 12.5 ng/µL in 25 mmol/L ABC. For the alpha-lytic WT protease solution, the protease was at 25 ng/µL in 25 mmol/L ABC. Samples were left on ice for 30 min for rehydration in the protease solution and then excess liquid was removed. Gel pieces were then quickly rinsed with 50 µL of 25 mmol/L ABC. Durations and temperatures of digestion were as previously described for the in-solution digestions. After digestion, peptide extraction was completed using 75 µL of 50% ACN containing a 5% volume fraction of formic acid (FA) (Thermo Fisher Scientific) with a 30 min incubation at room temperature followed by 5 min of sonication in an Elma S 300 H bath sonicator (Elma Schmidbauer GmbH, Singen, Germany). Extraction using 50% ACN containing a 5% volume fraction of FA was completed twice and extraction using 50% isopropanol (VWR Chemicals, Radnor, PA, USA) containing a 5% volume fraction of FA was completed once. After extraction, the resulting pool extract was dried down in a speedvac for 1 h and reconstituted in a 0.1% volume fraction of TFA for monospin purification.

Peptide digests were desalted with MonoSpin C18 solid-phase extraction spin columns (GL Sciences, Tokyo, Japan). Before sample loading, each column was activated twice with 200 µL of HPLC-grade acetonitrile (ACN), with centrifugation at 2300× g for 1 min after each addition, and then equilibrated twice with 200 µL of 0.1% TFA using the same centrifugation conditions. After the equilibration flowthrough was discarded, the digest was loaded and centrifuged at 2300× *g* for 2 min. The retained peptides were washed twice with 200 µL of 0.1% TFA, centrifuging at 2300× *g* for 1 min after each wash, and the wash fractions were discarded. Peptides were eluted by two sequential additions of 100 µL of 60% ACN containing a 0.5% volume fraction of formic acid, followed by two additions of 100 µL of 80% ACN containing a 0.5% volume fraction of formic acid; the same centrifugation conditions were used for each elution. The combined eluate was dried in HPLC mode at 40 °C for approximately 1 h, or until dry, using the Genevac EZ-2 SpeedVac. Samples were finally reconstituted in 28.5 µL of 2% ACN containing a 0.1% volume fraction of formic acid to obtain a peptide concentration of 175 ng/µL.

### 2.3. Instrumental Analysis

Samples were analyzed as previously reported [3] using an Orbitrap Fusion Lumos Tribrid mass spectrometer equipped with a Nanospray Flex ion source and coupled in-line to an Ultimate 3000 nanoLC HPLC system (Thermo Fisher Scientific). Between 1 µg and 5 µg of digested peptide material was loaded onto an Acclaim PepMap RSLC reversed-phase column (75 µm × 25 cm; Thermo Fisher Scientific). Chromatographic separation was performed over 230 min at 300 nL/min. The gradient was maintained at 2% ACN for the first 25 min, increased to 32% ACN by 142 min, increased further to 98% ACN by 165 min and held for 10 min, and then returned to 2% ACN at 180 min, where it remained through the end of the run. Full MS spectra were acquired in the Orbitrap from *m/z* 380 to 2000 at a resolution of 120,000, with a maximum ion injection time of 50 ms, an automatic gain control target of 400,000, and a radio-frequency lens setting of 40%. Data-dependent precursor selection was restricted to charge states 2–8 and intensities from 5 × 10^4^ to 1 × 10^20^, with a 15 s dynamic-exclusion period and a total cycle time of 5 s. Precursors were prioritized by higher charge state and lower *m/z*. Tandem mass spectra were collected after quadrupole isolation with a 2 *m/z* unit isolation window. Two Orbitrap MS2 acquisition modes were used: stepped fragmentation and contingent ion-trap fragmentation. For both modes, the maximum injection time was 60 ms, the automatic gain control target was 50,000, and the Orbitrap resolution was 30,000. The stepped method used higher-energy collisional dissociation (HCD) in the beam-type collision cell at normalized collision energies of 15%, 25%, and 35%. Detection of the HexNAc oxonium ion at *m/z* 204.087 in the HCD spectrum triggered ion-trap fragmentation, which was performed at 30% collision energy with an activation time of 10 ms. A complete list of raw data files acquired using the described method is presented in Table S2.

### 2.4. Data Processing

FASTA files were generated for vaccines and virus reagents based on reported protein sequence information from either GISAID [22] for influenza virus or from UniProt [23] for antigens from Shingrix and COVID-19 vaccines. See Table 1 for information on strains, lineages, and subtypes for each vaccine. Potential protein contaminants were also included in each FASTA file depending on the expression or propagation source. Proteins reported to be in high abundance in the hen egg proteome of *Gallus gallus* were included in all egg-based vaccine FASTA files. Fall armyworm and closely related insect species (*Bombyx mori*, *Drosophila melanogaster*, *Anopheles gambiae*, and *Spodoptera frugiperda*), as well as baculovirus proteins (*Alphabaculovirus aucalifornicae*) were included in Sf9-based vaccine FASTA files. Proteins in high abundance in the kidney of *Canis lupus familiaris* were included in all MDCK cell line-based vaccines. Proteins in high abundance in ovum cells of *Cricetulus griseus* were included in the Shingles vaccines FASTA file. See FASTA files provided in ProteomeXchange for more information. Note that viral glycoprotein sequences obtained from GISAID are not reported in these files for privacy obligations. However, associated EPI_SET ID for each vaccine is listed in Figure S1.

MSFragger version 4.1 output provided the primary glycopeptide identifications and abundance measurements [24,25]. Searches allowed up to three missed cleavages, except for alpha-lytic protease searches, for which up to six missed cleavages were permitted. Precursor and fragment mass tolerances were 10 ppm and 20 ppm, respectively. Carbamidomethylation of cysteine (+57.02 Da) was specified as a fixed modification, whereas methionine oxidation (+15.99 Da) and N-terminal ammonia loss from glutamine (−17.03 Da) were treated as variable modifications. Searches used a collection of 444 N-glycans assembled from mammalian, human, and sulfation glycan databases. Though a Sf9 and MDCK-specific database was not used, common N-glycans reported from these sources are included in this database. The same search space was applied to all samples to avoid source-dependent search bias. Conventional false-discovery-rate (FDR) filtering was not used as the final validation step. Instead, candidate glycopeptide identifications underwent orthogonal validation based on retention time, oxonium-ion and Y-ion evidence, HCD and ion-trap spectral consistency, precursor purity, XIC overlap, and the number of supporting HCD spectra.

Annotated tandem glycopeptide libraries were generated with the previously reported in-house software workflow [3]. MSFragger results supplied preliminary glycopeptide assignments and MS1 extracted-ion-chromatogram (XIC) information [26]. Tandem spectra for peptides and intact N-glycopeptides were annotated by sequence, precursor charge state, and modification using MS_Piano [27], and the spectra presented in this study used those annotations. Candidate glycopeptides were subsequently evaluated using contingent ion-trap collision-induced-dissociation spectra, chromatographic retention time, Y-ion evidence, spectral purity, XIC overlap, and the number of identified HCD spectra. Spectra that passed validation were converted to NIST MS library format. Generation of a GADS required at least two distinct, well-identified glycopeptides. The resulting GADS libraries were viewed, compared, and searched with NIST MS Search [28].

For each digest, site-specific glycosylation distributions were stored as glycopeptide abundance distribution spectra (GADS). A GADS combines the glycoforms observed for one peptide sequence in a single mass spectrometry injection. These pseudo-spectra were assembled from MS2 glycopeptide identifications by plotting glycan mass on the x-axis and the corresponding MS1 XIC abundance on the y-axis. Peaks shown in red denote lower-confidence glycopeptide assignments, which may result from factors such as inconsistent retention time, absence of a Y1 ion, or absence of expected oxonium ions. Each single-charge-state GADS therefore consolidates all detected glycopeptides for one sequence and one run, allowing glycan distributions for the same sequence to be compared across repeated analyses. When two or more charge-state-specific GADS were available for a sequence, a combined multiple-charge-state GADS was also constructed as described previously [29]. In-house software generated the GADS pseudo-spectra by integrating MSFragger output with validation information from the tandem mass spectral library workflow.

### 2.5. Statistical Analysis

Similarity between glycosylation distributions was quantified with the cosine-like scoring function used in NIST library searches, referred to in this study as the similarity score or dot product, as described by Stein et al. [13]. This score was used to rank GADS according to their resemblance. Whereas a conventional mass spectrum is defined by ion mass and abundance, a GADS uses glycan mass and MS1-derived glycopeptide abundance compiled from multiple glycopeptides [29]. Although combined multiple-charge-state GADS were generated, all statistical comparisons reported here used glycopeptides from a single precursor charge state. No comparison was assigned when a peptide or glycosylation site was detected in only one member of a sample pair. Variation among the similarity scores, interpreted as reflecting differences in glycosylation, was summarized with box-and-whisker and violin plots. For the box-and-whisker plots, the center line denotes the median, the box spans the interquartile range, and the whiskers extend to observations within 1.5 times the interquartile range unless stated otherwise. In violin plots, width represents the relative density of similarity scores. RStudio (version 2023.03.01 Build 446) was used to generate these plots and to construct hierarchical-clustering dendrograms from pairwise GADS similarity scores for identification of glycan-distribution classes (Figure S2) [30]. Similarly, a taxonomy-based tree of the production-host species was generated using PhyloT from the NCBI taxonomy available at https://phylot.biobyte.de/ (accessed on 12 November 2025) and visualized in the interactive tree of life webpage available at https://itol.embl.de/tree/ (accessed on 12 November 2025) [31,32].

## 3. Results

### 3.1. Summary of Glycosylation Distributions Across All Vaccines

In the analyses reported here, each GADS peak represents a glycan with monosaccharides represented as follows: G represents HexNAc (N-acetylhexosamine), H represents hexose, F represents fucose (deoxyhexose), S represents sialic acid (N-acetylneuraminic acid), and Po represents phosphorylation [29]. Site-specific glycosylation analysis in this study depended on both the number of glycopeptide spectral identifications and the ability to localize those identifications to individual glycosylation sites from the surrounding peptide sequence. Protein coverage is therefore reported in terms of glycopeptide spectral identifications and site-specific sequence coverage (Figure S3). Two glycoproteins were analyzed in influenza vaccines: hemagglutinin (HA) and neuraminidase (NA). HA is typically present at higher abundance than NA in influenza virions, often reported on the order of ≈ 7× to ≈ 10×, consistent with our findings of fewer NA spectral identifications, resulting in lower NA site coverage (Figure S3) [33]. For HA expressed from mammalian sources, the most prevalent glycans are complex glycans, such as G4H5 and G5H5, with and without core fucosylation and high-mannose glycans. In the same expression systems, NA more frequently exhibited distributions that were either predominantly high mannose or predominantly higher-mass complex glycans with antennary fucosylation, such as G5H6F3, G6H7F, and G6H7F2. Other glycoproteins measured in this work include the SARS-CoV-2 spike (S) protein produced by Sf9 cell recombinant methods and surface glycoprotein E (gE) from varicella-zoster virus produced in a CHO cell line. The S protein has 21 N-linked glycosylation sites, of which 20 were reproducibly measured in site-specific glycosylation, while gE has three N-linked glycosylation sites, of which two were reproducibly measured. The S protein had glycan distributions predominated by paucimannose and the gE protein was predominated by high-mass multi-sialylated complex glycans.

**Figure 2:**
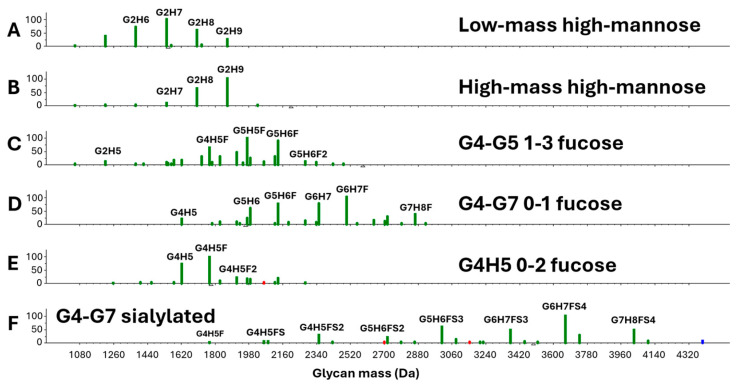
Common glycosylation distributions across all vaccines and reagent glycoproteins. Six GADS classes were common among all distributions measured for N-linked glycosylation sites. Glycan mass is represented on the X-axis and relative abundance is represented on the Y-axis. GADS used to exemplify the six classes were as follows: (**A**) low-mass high mannose distribution, as exemplified from HA in FCVX-22; (**B**) high-mass high-mannose distribution, as exemplified from HA in AFLQ-21; (**C**) G4–G5 1–3 fucose type distribution, as exemplified from HA in AFLQ-22; (**D**) G4–G7 0–1 fucose type distribution, as exemplified from HA in NIBSC-NC99; (**E**) G4H5 0–1 fucose type distribution, as exemplified from HA in NIBSC-NC99; and (**F**) G4–G7 0–4 sialylated type distribution from gE in SHGX. See Table 1 for a list of product abbreviations.

Many different GADS exist across our measured antigens (HA, NA, S, and gE), with numerous strains, subtypes, and lineages measured per protein for HA and NA. This yields thousands of reproducibly unique GADS across the dataset. However, six general classes of GADS were commonly observed. The first class is a high-mannose distribution with predominantly low-mass glycans such as G2H6 and G2H7 (Figure 2A). This distribution is indicative of less glyco-processing and is common in HA and NA, with most observations being in the head region of HA. The second class is a high-mannose distribution with predominantly high-mass glycans such as G2H8 and G2H9 (Figure 2B). This distribution is indicative of slightly less glyco-processing than the low-mass distribution and is common in HA. Similar to the low-mass distribution, it is frequently observed in the HA head region. The third and fourth classes of GADS are multi-fucosylated complex distributions with four to seven HexNAc (Figure 2C,D). These types of distributions are observed in both the head and stalk regions of HA. The fifth class is a relatively simple distribution of G4H5 with varying degrees of fucosylation (Figure 2E), also found in the head and stalk regions of HA. Finally, the sixth class is a distribution of multi-sialylated complex glycans that, in this dataset, is unique to the gE protein produced in CHO cells (Figure 2F). While these six classes were commonly identified, blended distributions also occurred, such as a combination of high-mannose and low-mass complex glycans in the HA stalk (Figure S4). Previous work characterizing site-specific glycosylation profiles in recombinant HA and NA proteins expressed from the HEK293 cell line has also described major classes of glycan distributions [3]. Compared to the distributions emphasized here, those HEK293 profiles were reported to be primarily sialylated and mono-fucosylated bi-antennary complex glycans such as G4H3F, G4H4F, and G4H5FS.

**Figure 3:**
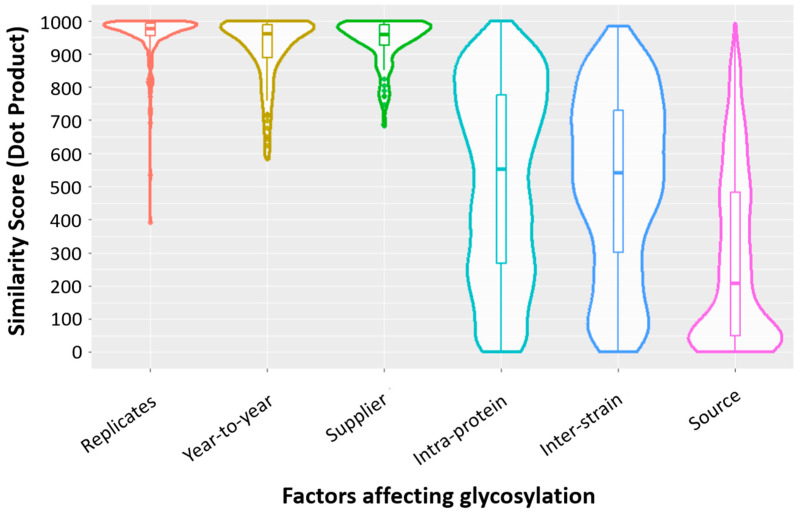
Violin plot of glycosylation similarity based on different factors of comparison. Measured factors that influence variation in glycosylation distribution include replicates (injection and digestion replicates combined), inter-annual vaccine production, different suppliers of the same vaccine virus component, different glycosylation sites on the same protein, homologous and non-homologous glycosylation sites from different strains, subtypes, and lineages within the same vaccine, and different production sources of the same virus component in different vaccines. In box-and-whisker plots, the center line represents the median, boxes indicate the interquartile range, and whiskers extend to values within 1.5× the interquartile range; in violin plots, plot width indicates the relative density of similarity scores.

GADS comparisons were made quantitatively using a dot-product similarity metric implemented in NIST MS Search [34]. Except for intra-protein comparisons, all GADS similarity analyses were performed only between matched glycopeptides representing the same glycosylation site, peptide sequence, and precursor charge state so that differences in score primarily reflected glycan-distribution differences rather than peptide-level differences. A summary of GADS similarity across the factors evaluated is provided in Figure 3. See Table S3 for a breakdown of comparisons. For replicates, GADS comparisons included injection replicates and digestion replicates. Injection- and digestion-replicate groups had similar score distributions with the majority of data from both distributions overlapping (Figure S5). Overall, replicate comparisons had a median similarity score of 978. This demonstrates the high degree of measurement reproducibility attainable by the present method. Note that a similarity score above 900 is generally considered to be excellent agreement, scores between 800 and 900 are considered to be good agreement, scores between 600 and 800 are considered moderate agreement, and scores below 600 are considered to be in low agreement [35,36]. The next comparison was between conserved influenza vaccine virus components present in different annual formulations for influenza quadrivalent vaccines, which had a very high median similarity score of 961. Also showing high similarity, different suppliers of the same virus component of influenza virus from the same production source had a median similarity score of 960. In this dataset, year-to-year production lots within the same supplier and supplier-to-supplier comparisons for egg-based influenza vaccines were therefore close to the similarity observed for digestion and injection replicates. The other three GADS comparisons had lower similarity: intra-protein comparisons (median score = 554), inter-strain comparisons for influenza vaccines (median score = 540), and the same strain of influenza produced in a different production source (median score = 209). These lower similarities illustrate the selectivity of this method for differentiating biologically meaningful sources of glycosylation variation. These three factors are especially interesting as different glycosylation sites on the same protein can have distinct GADS, different strains can differ in sequence and structure, and the production source has a strong influence on glycosylation.

### 3.2. Year-to-Year Variation in Glycosylation

**Figure 4:**
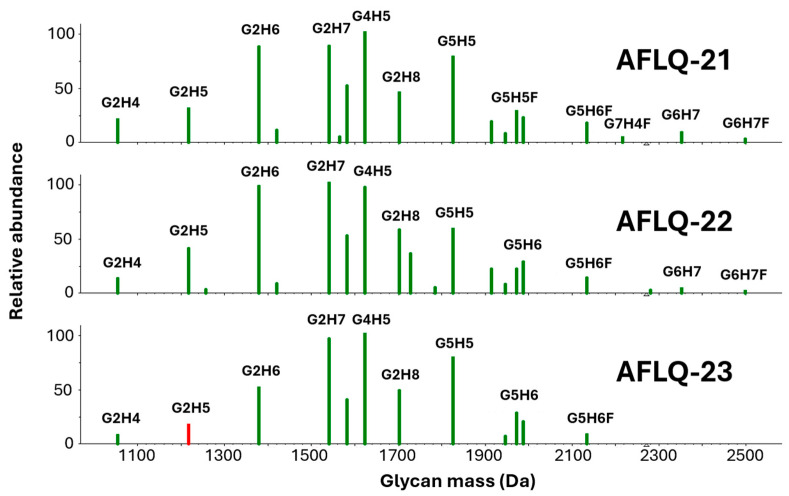
Year-to-year variation in glycosylation among three annual formulations of an influenza vaccine. This figure exemplifies the year-to-year factor of Figure 3. These are glycopeptide abundance distribution spectra (GADS) comparisons between the same site of the same protein from the same virus component which was conserved between three production years for AFLQ-21, AFLQ-22, and AFLQ-23. Peaks shown in red denote lower-confidence glycopeptide assignments. See methods Section 2.4 for more information on red colored peaks.

Year-to-year variation in GADS is the measurement of changes in glycan distributions due to differences in formulations from the same supplier. This metric is limited in this work to influenza quadrivalent vaccines, as these were the only vaccines obtained that contain different year-to-year formulation. Specifically, this includes two virus components between AFLQ-21 and AFLQ-22; three virus components between AFLQ-22 and AFLQ-23; and three virus components between FCVX-22 and FCVX-23. See Table 1 for more details on these virus components, sometimes referred to in this work as strains. Across these conserved-strain comparisons, the spread in similarity scores was small (Figure 3). Most values below a similarity score of 900 were associated with GADS built from fewer identifications and/or GADS with broader apparent glycan dropout. Figure 4 provides an example of a glycosylation site measured consistently between AFLQ-21, AFLQ-22, and AFLQ-23 formulations. B/Phuket/3073/2013 is present in all three formulations and shows a highly consistent glycosylation distribution at this site. The N-glycans with the most variability between year-to-year formulations in this example include G2H7, G2H8, and G5H5F. Overall, this illustrates the reproducibility of both site-specific glycosylation methods and of biomanufacturing vaccines over many different yearly formulations.

### 3.3. Supplier Variation in Glycosylation

To isolate supplier-to-supplier differences, we compared GADS within egg-produced vaccines for related influenza virus components. Results indicate high consistency in glycosylation between different suppliers of egg-based quadrivalent influenza vaccines, with a median similarity score of 960 (Figure 3). GADS with similarity above 980 in this category were predominantly high-mannose-type distributions. GADS with similarity below 900 more often contained complex glycans with four to seven HexNAc and one to three fucose residues. Outliers with similarity scores below 800 were typically associated with longer and higher charge state peptides and low numbers of peptide identifications.

**Figure 5:**
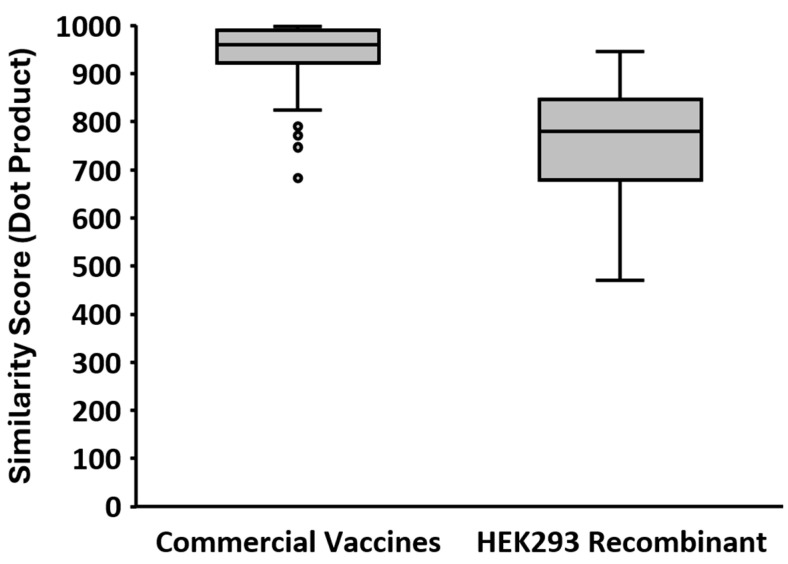
Supplier variation in glycosylation from commercial influenza vaccines vs HEK293 recombinant influenza glycoproteins. This figure expands the supplier-comparison category presented in Figure 3. This box and whisker plot demonstrates the difference in glycosylation distribution between commercial influenza vaccines and HEK293 recombinant influenza glycoproteins from other work [3]. Similarity scores include values from both hemagglutinin and neuraminidase. The center line represents the median, boxes indicate the interquartile range, and whiskers extend to values within 1.5× the interquartile range.

The similarity of glycosylation distributions was higher between suppliers producing egg-based influenza vaccines than recombinant proteins expressed in HEK293 cells. Previous work applied the same types of site-specific glycosylation comparisons using the same analytical and data-analysis approaches used here [3]. Across those datasets, the median similarity score for the egg-based vaccines was 960, whereas the median score for the HEK293-produced versions was 781 (Figure 5). The interquartile range for the egg-based vaccines was 922–990, whereas the interquartile range for the HEK293 influenza proteins was 679–847. Together, these results show that, under the measurement and analysis conditions used, egg-based production yielded more similar GADS across compared sites than the HEK293 recombinant proteins in the referenced dataset. Note that influenza strains (i.e., viral components) were not identical between these two groups; however, the glycoproteins and methods for measurement and analysis were consistent.

### 3.4. Intra-Protein and Inter-Strain Variation in Glycosylation

Variation in site-specific glycosylation across different sites within the same glycoprotein (meta-heterogeneity) reflects the overall diversity of glycan processing across a protein and can help define how local structural and biosynthetic constraints differ between regions. In this work, intra-protein similarity was highest for the Sf9-derived proteins and more heterogeneous for the egg-derived influenza vaccine proteins (Figure S6), consistent with the narrower glycan repertoire observed in the insect-cell system. Within HA from influenza A and B, the stalk region showed the greatest internal concordance, with fucosylated complex-type distributions enriched in the stalk, whereas head-region sites more often displayed high-mannose-enriched distributions (Figures S7–S10). We also assessed inter-strain variation to determine the extent to which different virus components of the same vaccine retain similar site-specific glycosylation at homologous regions despite differences in primary sequence. In this work, inter-strain is defined as comparing the same protein (e.g., HA) across the four virus components of an influenza vaccine, including the two influenza A subtypes and two influenza B lineages. This comparison is especially relevant in seasonal influenza vaccines, which combine multiple related but non-identical viral components (i.e., strains) in a single formulation. Quadrivalent influenza vaccines contain four virus strains: two influenza A strains representing the H1N1 and H3N2 subtypes, and two influenza B strains representing the Victoria and Yamagata lineages. While these four strains differ in antigen protein primary sequence, they retain regions with related structure and sequence. Such conserved regions can also share functional features; for example, glycan-binding and structural studies have shown that HA can engage bi-antennary glycans terminating with α-2,6 linked sialic acids [37]. The goal of this comparison was to assess GADS similarity within the same vaccine across all virus components for both HA and NA. Overall, the median GADS similarity score for this factor was 540 with an interquartile range of 293 to 723 (Figure 3). These comparisons are limited to quadrivalent influenza vaccines and were performed between two categories of sites: homologous sites between proteins and non-homologous sites. Homologous regions were determined using sequence alignment, and the resulting alignments between the four influenza strains are shown in Figures S11 and S12.

**Figure 6:**
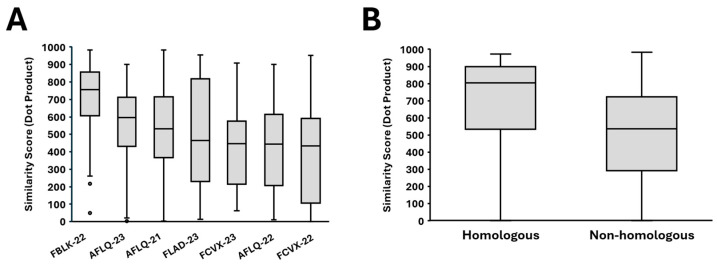
Variation in glycosylation between different influenza virus components within the same vaccine. For box-and-whisker plots, the center line represents the median, boxes indicate the interquartile range, and whiskers extend to values within 1.5× the interquartile range. (**A**) Box and whisker plot of glycosylation similarity between non-homologous sites of different virus components within the same vaccine. (**B**) Box and whisker plot of glycosylation similarity between homologous and non-homologous regions of all influenza vaccines. Homologous regions were determined using sequence alignment as seen in Figures S11 and S12.

Results indicate a high degree of inter-strain glycosylation similarity within FBLK-22 and moderate similarity in egg-based and MDCK cell line-produced vaccines (Figure 6). Median similarity scores for inter-strain comparisons for all influenza vaccines include 755 for FBLK-22, 595 for AFLQ-23, 532 for AFLQ-21, 464 for FLAD-23, 446 for FCVX-23, 444 for AFLQ-22, and 433 for FCVX-22 (Figure 6). As noted previously, a major driver of GADS similarity is the production platform or expression system. Here, the Sf9 recombinant vaccine showed the highest similarity, consistent with its lower diversity of glycosylation distributions, which are primarily composed of high-mannose and paucimannose glycans. The remaining quadrivalent influenza vaccines from egg-based and MDCK cell line sources include mixtures of high-mannose distributions and multiple classes of complex glycans and therefore show lower similarity. When stratifying by homologous versus non-homologous site comparisons, homologous regions had higher similarity (median = 803) than non-homologous regions (median = 536). This is consistent with prior studies comparing different strains or subtypes of viral glycoproteins and may reflect conservation of both sequence and structural context in homologous regions [3,16,38].

### 3.5. Source Variation in Glycosylation

**Figure 7:**
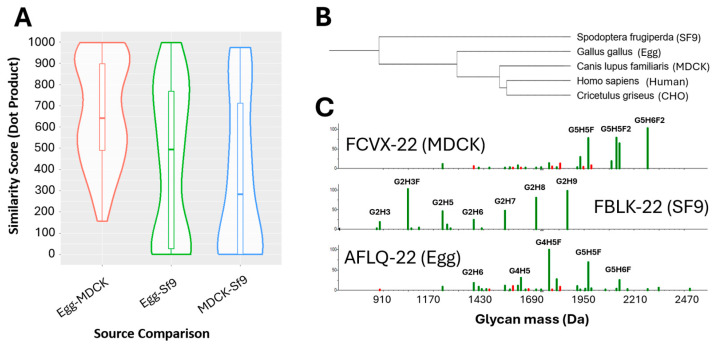
Variation in glycosylation between different production sources. This figure expands the production-source comparison category presented in Figure 3. In box-and-whisker plots, the center line represents the median, boxes indicate the interquartile range, and whiskers extend to values within 1.5× the interquartile range; in violin plots, plot width indicates the relative density of similarity scores. (**A**) Violin plot representing all source comparisons. Only influenza virus glycoproteins are included here. Note that the peptide, charge state, amino acid position, strain, and protein are all equivalent between similarity comparisons. (**B**) Phylogenetic tree indicating evolutionary relationships between the four production sources, along with *Homo sapiens* as a reference. (**C**) Visual comparison of glycosylation distribution similarities between the MDCK, Sf9, and egg-based production sources for an equivalent glycosylation site between the three vaccines.

The largest contributor of glycosylation pattern change evaluated in this work was the production source of the vaccine. It is well established that the biological host used for protein production can strongly influence post-translational modifications and glycosylation patterns [9,14,39]. Here, production sources were compared quantitatively in terms of GADS similarity. The workflow described here is the first instance of a quantitative comparison of glycosylation similarity in an interpretable and easily searchable format. This source comparison was intentionally limited to cases where the same proteins from the same viral components were available across multiple production sources. Accordingly, comparisons were restricted to quadrivalent influenza vaccines from egg-based sources, MDCK cell line propagation, and Sf9 recombinant protein expression. Among the two-way comparisons, egg-based vaccines versus the MDCK cell line vaccine showed the highest similarity, with a median similarity score of 641 (Figure 7A). The next highest two-way comparison was egg-based vaccines versus the Sf9 recombinant vaccine, with a median similarity score of 494. Finally, the lowest similarity was observed for the MDCK cell line vaccine versus the Sf9 recombinant vaccine.

Quantitative GADS similarity between production sources can be useful in comparability and manufacturing-context studies, particularly when glycosylation microheterogeneity is expected to influence biophysical properties or function for a given product [5,9]. As site-specific methods and high-resolution mass spectrometry have matured, it has become increasingly feasible to resolve and compare glycan compositions that differ by small mass increments using stepped-energy fragmentation approaches. In the results described here, the two vertebrate-based production platforms were the most similar in glycosylation. We therefore performed a taxonomic comparison to evaluate whether broad relatedness among the production hosts was consistent with the observed ordering of glycosylation similarity. In this dataset, the broad taxonomic relationships represented in the PhyloT tree are consistent with the ordering of GADS similarity (Figure 7A,B). At the glycan level, GADS similarity across sources was largely associated with differences in the presence and relative abundance of fucosylated complex glycans: egg-based influenza vaccines produced more GADS with lower-mass glycans such as G4H5, G4H5F, and G5H5F, whereas the MDCK cell line produced higher-mass complex glycans with more fucosylation such as G5H5F, G5H5F2, and G5H6F2 (Figure 7C).

## 4. Discussion

Site-specific glycosylation analysis, though not required for vaccine release or regulatory submission, can provide useful orthogonal characterization when developers need to document microheterogeneity, compare products or manufacturing changes, or investigate process drift. The principal contribution of this work is that glycan distributions at individual sites can be recorded in a standardized, spectrum-like format and compared quantitatively across relevant biomanufacturing scenarios. Using this framework, we demonstrate the utility of site-specific glycosylation profiling for (i) defining overall classes of glycosylation, (ii) quantitatively reporting similarity across different reproducibility scenarios, and (iii) assessing how glycosylation patterns vary as a function of biological production source. Across the datasets analyzed, we observe high similarity in glycosylation patterns between replicate measurements, year-to-year production of the same antigen, and different suppliers producing the same antigen, whereas similarity is lower between different glycosylation sites on the same protein, the same antigen from different virus components, and different biological production sources (Figure 3).

A key methodological contribution of this study is the application of glycopeptide abundance distribution spectra (GADS) as a practical representation for storing, visualizing, and comparing site-specific glycosylation distributions in vaccines. The GADS method incorporates rigorous qualifications for glycopeptide identification, including the assessment of multi-fucosylated glycopeptides (Figure S13) [3,29]. Other groups have made quantitative comparisons between site-specific glycosylation measurements, but many approaches are less amenable to library-based quality assurance and quality control workflows and to comparisons across non-matching sites [40]. The GADS representation described in our previous work enables fast, interpretable comparisons between glycosylation sites without relying on class-level summaries such as pie charts [3,29,38,41]. In addition, the NIST MS Search library-search paradigm supports quantitative comparison across large numbers of GADS, including cases where sites are not known a priori to be similar or dissimilar relative to a reference set. For vaccine developers, this offers a practical way to archive site-specific glycosylation measurements, compare new measurements against prior lots or reference materials, and identify where differences arise between different conditions of the biomanufacturing process.

Influenza vaccines provide a stringent test case for this type of analysis because they contain two major glycoprotein antigens, HA and NA, across multiple viruses of different type/subtype/lineage and manufacturing platforms. Many site-specific glycosylation studies focus on single purified proteins with relatively few glycosylation sites, whereas quadrivalent influenza vaccines present a much larger analytical burden, with on average approximately 70 glycosylation sites across the four viral components for HA and NA (Figure S3). The present results show that high-resolution mass spectrometry workflows can be applied at this scale and can generate site-specific distributions across commercially relevant products. This demonstrates that detailed glycosylation recording is feasible even for compositionally complex products. More broadly, comprehensive glycoprotein detection may support quality assurance by helping detect process drift affecting protein modification patterns and by providing an additional analytical layer when manufacturing changes are evaluated [42,43].

In our measurements, replicates, year-to-year production, and different suppliers of the same antigen had a high degree of similarity (Figure 3, Figure 4 and Figure 5). The replicate similarity is consistent with previous work and supports the analytical and data-analysis workflow used here [3]. Lower-scoring comparisons were largely associated with GADS built from relatively few glycopeptide identifications, indicating that undersampling can reduce confidence and inflate apparent differences. In practice, this means that identification depth and filtering criteria should be treated as part of the measurement specification when GADS are used for lot-to-lot or cross-platform comparison. For mature platforms such as egg-based influenza vaccine manufacturing, the observed consistency is also biologically plausible, given long-standing process optimization and standardization [44], whereas recombinant systems may be more vulnerable to biological variability [45,46]. Continued quantitative characterization of glycosylation variability across production systems may therefore become increasingly relevant as recombinant vaccine products continue to expand [47].

Factors with intermediate glycosylation similarity included comparisons between different sites on the same protein and between different influenza types (e.g., HA from influenza A vs. influenza B). It is well established that different sites on the same protein can display distinct glycosylation patterns [48]. It has also been proposed that local structural context can modulate enzyme access during ER–Golgi processing, potentially contributing to similar glycosylation profiles between sites in close proximity [49]. Here, we found that GADS similarity did not correlate with either the amino-acid distance or the estimated spatial distance between glycosylation sites (Figure S14). In contrast, similarity was associated with protein domain. Specifically, glycosylation was more similar among sites in the stalk region than among sites in the head region of HA. This pattern is consistent with differences in local accessibility and processing along the secretory pathway; for example, some head-region sites may retain higher proportions of high-mannose glycans, reflecting limited processing. We also observed that glycosylation patterns among sites on NA (head and stalk) were, on average, more dissimilar than those on HA. This could reflect greater variability in steric accessibility and/or processing across NA sites [50]. More speculatively, site-to-site differences on NA could also be shaped by strain-specific constraints and evolutionary pressures acting at individual sites (e.g., HA-NA functional balance and NA stalk-length effects) [51,52,53]. In terms of influenza type, subtype, and lineage similarity, homologous regions between the four virus components within a vaccine were substantially more similar than non-homologous regions (Figure 6B), consistent with previous work [3]. For egg-based vaccines, we observed greater glycosylation similarity across different virus components in AFLQ-23 and AFLQ-21 formulations compared to the others; however, the distributions largely overlapped, and apparent differences may reflect the extent of sites shared between virus components rather than systematic formulation effects.

Production source had the largest effect on glycosylation profile (Figure 3 and Figure 7), making it the clearest example of where a standardized site-specific comparison framework may be useful in vaccine development. While not studied here, it has been shown that other aspects of the production pipeline (e.g., bioprocessing stage) also have a large effect on glycosylation and other post-translational modifications [54,55]. For alternative routes of vaccine production, such as recombinant expression in insect cell lines or viral propagation in MDCK cells, site-specific glycosylation differences have been reported in other work, but the magnitude of variation across large numbers of sites has not been adequately quantified. Here, we observed that glycosylation similarity between production sources broadly tracks phylogenetic relatedness among the source species (Figure 7A,B) in the sense that more closely related hosts tend to yield more similar site-specific distributions. Production-source effects also likely contribute to the comparatively high similarity between intra-protein sites and inter-strain sites in the Sf9 recombinant vaccines (Figure 6 and Figure S6), insofar as a shared host-processing environment can dominate over sequence- or site-specific differences. Finally, the distinct glycosylation profiles observed for the CHO-produced Shingrix antigen (Figure 2F) should be interpreted in the context of identity (gE versus HA/NA), site occupancy, and processing context.

There are several limitations for applying site-specific glycosylation mass spectrometry to vaccine characterization. First, quadrivalent influenza vaccines pose a proteomics challenge because the HA and NA from four viruses have substantial sequence homology (Figures S11 and S12). With our current methods, 31% of HA glycosylation sites and 8% of NA glycosylation sites were indistinguishable. One strategy to increase differentiation among homologous regions is to use proteases that generate longer peptides, increasing the chance of including distinguishing residues. However, longer peptides can also increase the frequency of multiply glycosylated peptides, which is less amenable to site-specific analysis. Second, intact-glycopeptide analysis is analytically more complex than released-glycan glycomics because signals can be distributed among multiple peptide–glycan combinations, proteolytic forms, and precursor charge states. In addition, confident identification requires assignment of both the peptide and glycan components and localization to a specific glycosylation site. Consequently, sampling depth and coverage of individual glycosites and low-abundance glycoforms may be incomplete. This limits the dynamic range for detecting low-abundance glycopeptides, as exemplified in NA in this work. Third, many vaccines contain adjuvants or excipients that require cleanup due to incompatibility with digestion workflows and/or LC–MS analysis. In this study, we used an in-gel digestion protocol for adjuvanted vaccines, which can reduce recoveries and contributed to fewer glycopeptide identifications for some samples. Thus, while adjuvants do not preclude site-specific measurements, they can impede sensitivity and coverage. Future studies could evaluate alternative cleanup strategies to minimize losses of glycoprotein material while maximizing removal of interfering components.

Additional limitations arise from data processing and glycan structure interpretation. In terms of data processing, the glycan database used could limit the comparison to only patterns that are common among all sources. The search space in this work included the major conventional N-glycan compositions expected from mammalian, avian, and Sf9 production systems but was not designed to capture every host-specific glycan modification. Cross-source GADS comparisons therefore quantify the detected composition-level abundance distributions rather than complete host-specific structural glycomes. In terms of structural interpretation, the present intact-glycopeptide workflow primarily resolves glycans at the monosaccharide-composition level and does not routinely distinguish structural or linkage isomers with identical precursor masses. Although glycan fragment ions can sometimes provide evidence for specific substructures, the data generally do not support comprehensive or unambiguous isomer assignment.

## 5. Conclusions

This work provides a practical way to record and compare mass spectrometry-based site-specific glycosylation distributions in protein subunit and inactivated virus vaccines using a spectrum-like GADS representation and quantitative similarity scoring. This method is not only valuable for descriptive glycoprofiling, but is a reusable framework for documenting microheterogeneity, assessing analytical precision, and comparing products across lots, suppliers, strains, and production platforms. Across the vaccine set analyzed, we report glycosylation distributions for monovalent and quadrivalent influenza vaccines, a SARS-CoV-2 vaccine, and a varicella-zoster vaccine. Our framework showed high similarity where comparability would be expected (i.e., replicates, year-to-year conserved viral components, and equivalent production-source suppliers) and lower similarity where biological or process differences were expected to be larger, especially across production sources. Thus, our hypothesis that GADS-based scoring could provide reproducible and informative quantitative comparisons of site-specific glycosylation profiles was supported. While portions of these findings have been reported elsewhere, the present study provides a unified quantitative evaluation of similarity using a common GADS-based framework on complex commercial products. We also report a quantitative concordance between broad phylogenetic relatedness of production hosts and glycosylation similarity across sources. Finally, although glycosylation can influence biological function for some antigens, the present work is focused on analytical characterization and comparability; linking site-specific glycosylation distributions to vaccine immunogenicity or efficacy will require dedicated functional studies.

## Figures and Tables

**Table 1:** List of vaccines obtained for site-specific glycosylation LC-MS/MS analysis.

Vendor/Vaccine	Abbreviation	Virus	Antigen(s)	Strain/Lineage/Subtype	Source
NIBSC	NIBSC-NC99	Influenza A	Hemagglutinin & Neuraminidase	A/New Caledonia/20/1999	Egg (Inactivated)
NIBSC	NIBSC-PH82	Influenza A	Hemagglutinin & Neuraminidase	A/Philippines/2/1982	Egg (Inactivated)
NIBSC	NIBSC-SW13	Influenza A	Hemagglutinin & Neuraminidase	A/Switzerland/9715293/2013	Egg (Inactivated)
Creative Biomart	CB-PA99	Influenza A	Hemagglutinin & Neuraminidase	A/Panama/2007/1999	Egg (Inactivated)
Creative Biomart	CB-NC99	Influenza A	Hemagglutinin & Neuraminidase	A/New Caledonia/20/1999	Egg (Inactivated)
Creative Biomart	CB-SD93	Influenza A	Hemagglutinin & Neuraminidase	A/Shandong/9/1993	Egg (Inactivated)
Afluria Quadrivalent 2021	AFLQ-21	Influenza A & Influenza B	Hemagglutinin & Neuraminidase	A/Victoria/2570/2019 A/Cambodia/e0826360/2020 B/Victoria/705/2018 B/Phuket/3073/2013	Egg (Inactivated)
Afluria Quadrivalent 2022	AFLQ-22	Influenza A & Influenza B	Hemagglutinin & Neuraminidase	A/Victoria/2570/2019 A/Darwin/6/2021 B/Austria/1359417/2021 B/Phuket/3073/2013	Egg (Inactivated)
Afluria Quadrivalent 2023	AFLQ-23	Influenza A & Influenza B	Hemagglutinin & Neuraminidase	A/Victoria/4897/2022 A/Darwin/6/2021 B/Austria/1359417/2021 B/Phuket/3073/2013	Egg (Inactivated)
Flublok Quadrivalent 2022	FBLK-22	Influenza A & Influenza B	Hemagglutinin	A/Wisconsin/588/2019 A/Darwin/6/2021 B/Austria/1359417/2021 B/Phuket/3073/2013	SF9 (Subunit)
Flucelvax Quadrivalent 2022	FCVX-22	Influenza A & Influenza B	Hemagglutinin & Neuraminidase	A/Delaware/55/2019 A/Darwin/11/2021 B/Singapore/WUH4618/2021 B/Singapore/INFTT-16-0610/2016	MDCK (Inactivated)
Flucelvax Quadrivalent 2023	FCVX-23	Influenza A & Influenza B	Hemagglutinin & Neuraminidase	A/Georgia/12/2022 A/Darwin/11/2021 B/Singapore/WUH4618/2021 B/Singapore/INFTT-16-0610/2016	MDCK (Inactivated)
Fluad Quadrivalent 2023	FLAD-23	Influenza A & Influenza B	Hemagglutinin & Neuraminidase	A/Victoria/4897/2022 A/Darwin/6/2021 B/Austria/1359417/2021 B/Phuket/3073/2013	Egg (Inactivated)
Novavax COVID-19 2023	NVX-23	SARS-CoV-2	Spike Protein	Omicron XBB.1.5	SF9 (Subunit)
Shingrix	SHGX	Varicella-zoster	Surface Glycoprotein E	N/A	CHO (Subunit)

## Data Availability

The original data presented in the study are openly available on ProteomeXchange at https://www.proteomexchange.org/ (accessed on 2 April 2026) under identifier PXD074672. Datafiles are deposited and stored in the PRIDE partner repository at https://www.ebi.ac.uk/pride/ (accessed on 2 April 2026) [56,57]. A complete list of data files is presented in Table S2. GADS and MS^2^ libraries are freely available for download at Chemdata.nist.gov (accessed on 2 April 2026) [58] which can be visualized using NIST MS Search, which is also available for download at https://chemdata.nist.gov/dokuwiki/doku.php?id=chemdata:downloads:start#nist_ms_search_program (accessed on 2 April 2026) [28].

## Supplementary Materials

The following supporting information can be downloaded at: https://www.mdpi.com/article/10.3390/vaccines14070644/s1, Table S1: Supplier Information; Figure S1: Tables of EPISET identification numbers used to assemble FASTA files; Figure S2: Pairwise comparisons of all good quality GADS; Figure S3: Coverage of detection for all known glycosylation sites in acquired vaccines; Figure S4: GADS demonstrating a mixture of common classes; Table S2: List of raw files uploaded to repository and corresponding information; Figure S5: Box-and-whisker plot of replicate injections and replicate digestions; Figure S6: Intra-protein variation in glycosylation distribution within each vaccine; Figure S7: Correlation between glycosylation distribution similarity and site position from different sites on the same protein; Figure S8: Box-and-whisker plot of intra-protein variation within neuraminidase and hemagglutinin; Figure S9: GADS comparison of the HA head region in Afluria 2022–2023 formulation; Figure S10: GADS comparison of the HA stalk region in A/New Caledonia/20/1999; Figure S11: Glycosylation site alignment for hemagglutinin among quadrivalent influenza vaccines; Figure S12: Glycosylation site alignment for neuraminidase among quadrivalent influenza vaccines; Figure S13: Illustration of verification of multiply-fucosylated glycopeptide spectra; Figure S14: GADS similarity as a function of glycosylation site distance; Table S3: Summary of all glycosylation comparisons made in this study at the protein level.

## Abbreviations

The following abbreviations are used in this manuscript:

LC-MS/MS	Liquid chromatography tandem mass spectrometry
MDCK	Madin-Darby canine kidney
Sf9	*Spodoptera frugiperda* clone 9
CHO	Chinese hamster ovary
HA	Hemagglutinin
NA	Neuraminidase
GADS	Glycopeptide abundance distribution spectrum

## References

[1] An H.J., Froehlich J.W., Lebrilla C.B. (2009). Determination of glycosylation sites and site-specific heterogeneity in glycoproteins. Curr. Opin. Chem. Biol..

[2] Zaia J. (2008). Mass spectrometry and the emerging field of glycomics. Chem. Biol..

[3] Goecker Z.C., Burke M.C., Remoroza C.A., Liu Y., Mirokhin Y.A., Sheetlin S.L., Tchekhovskoi D.V., Yang X., Stein S.E. (2024). Variation of site-specific glycosylation profiles of recombinant influenza glycoproteins. Mol. Cell Proteom..

[4] De Leoz M.L.A., Duewer D.L., Fung A., Liu L., Yau H.K., Potter O., Staples G.O., Furuki K., Frenkel R., Hu Y. (2020). Nist interlaboratory study on glycosylation analysis of monoclonal antibodies: Comparison of results from diverse analytical methods. Mol. Cell. Proteom..

[5] Renner T.M., Stuible M., Rossotti M.A., Rohani N., Cepero-Donates Y., Sauvageau J., Deschatelets L., Dudani R., Harrison B.A., Baardsnes J. (2025). Modifying the glycosylation profile of SARS-CoV-2 spike-based subunit vaccines alters focusing of the humoral immune response in a mouse model. Commun. Med..

[6] Murakami M., Kiuchi T., Nishihara M., Tezuka K., Okamoto R., Izumi M., Kajihara Y. (2016). Chemical synthesis of erythropoietin glycoforms for insights into the relationship between glycosylation pattern and bioactivity. Sci. Adv..

[7] International Council for Harmonisation of Technical Requirements for Pharmaceuticals for Human Use (ICH) (1999). Q6B Specifications: Test Procedures and Acceptance Criteria for Biotechnological/Biological Products.

[8] U.S. Food and Drug Administration Current Good Manufacturing Practice for Finished Pharmaceuticals: Testing and Release for Distribution, 21 CFR § 211.165. https://www.ecfr.gov/current/title-21/chapter-I/subchapter-C/part-211.

[9] de Vries R.P., Smit C.H., de Bruin E., Rigter A., de Vries E., Cornelissen L.A., Eggink D., Chung N.P., Moore J.P., Sanders R.W. (2012). Glycan-dependent immunogenicity of recombinant soluble trimeric hemagglutinin. J. Virol..

[10] Maciola A.K., Pietrzak M.A., Kosson P., Czarnocki-Cieciura M., Smietanka K., Minta Z., Kopera E. (2017). The length of n-glycans of recombinant h5n1 hemagglutinin influences the oligomerization and immunogenicity of vaccine antigen. Front. Immunol..

[11] Monto A.S., Petrie J.G., Cross R.T., Johnson E., Liu M., Zhong W., Levine M., Katz J.M., Ohmit S.E. (2015). Antibody to influenza virus neuraminidase: An independent correlate of protection. J. Infect. Dis..

[12] Vainauskas S., Guntz H., McLeod E., McClung C., Ruse C., Shi X., Taron C.H. (2022). A broad-specificity o-glycoprotease that enables improved analysis of glycoproteins and glycopeptides containing intact complex o-glycans. Anal. Chem..

[13] Stein S.E., Scott D.R. (1994). Optimization and testing of mass spectral library search algorithms for compound identification. J. Am. Soc. Mass. Spectrom..

[14] An Y., Parsons L.M., Jankowska E., Melnyk D., Joshi M., Cipollo J.F. (2019). N-glycosylation of seasonal influenza vaccine hemagglutinins: Implication for potency testing and immune processing. J. Virol..

[15] Zost S.J., Parkhouse K., Gumina M.E., Kim K., Perez S.D., Wilson P.C., Treanor J.J., Sant A.J., Cobey S., Hensley S.E. (2017). Contemporary h3n2 influenza viruses have a glycosylation site that alters binding of antibodies elicited by egg-adapted vaccine strains. Proc. Natl. Acad. Sci. USA.

[16] Watanabe Y., Allen J.D., Wrapp D., McLellan J.S., Crispin M. (2020). Site-specific glycan analysis of the SARS-CoV-2 spike. Science.

[17] James S.F., Chahine E.B., Sucher A.J., Hanna C. (2018). Shingrix: The new adjuvanted recombinant herpes zoster vaccine. Ann. Pharmacother..

[18] Centers for Disease Control and Prevention 2024–2025 Flu Season. https://www.cdc.gov/flu/season/2024-2025.html.

[19] She Y.M., Li X., Cyr T.D. (2019). Remarkable structural diversity of n-glycan sulfation on influenza vaccines. Anal. Chem..

[20] Jimenez C.R., Huang L., Qiu Y., Burlingame A.L. (2001). In-gel digestion of proteins for maldi-ms fingerprint mapping. Curr. Protoc. Protein Sci..

[21] Thermo Fisher Scientific (2025). Nupage™ Technical Guide; MAN0000723.

[22] Shu Y., McCauley J. (2017). Gisaid: Global initiative on sharing all influenza data—From vision to reality. Euro Surveill..

[23] The UniProt Consortium (2025). Uniprot: The universal protein knowledgebase in 2025. Nucleic Acids Res..

[24] Kong A.T., Leprevost F.V., Avtonomov D.M., Mellacheruvu D., Nesvizhskii A.I. (2017). Msfragger: Ultrafast and comprehensive peptide identification in mass spectrometry-based proteomics. Nat. Methods.

[25] Polasky D.A., Yu F., Teo G.C., Nesvizhskii A.I. (2020). Fast and comprehensive n- and o-glycoproteomics analysis with msfragger-glyco. Nat. Methods.

[26] Wang G., Zhang Z., Liu Y., Burke M.C., Sheetlin S.L., Stein S.E. (2024). An xic-centric strategy for improved identification and quantification in proteomic data analyses. J. Proteome Res..

[27] Yang X., Neta P., Mirokhin Y.A., Tchekhovskoi D.V., Remoroza C.A., Burke M.C., Liang Y., Markey S.P., Stein S.E. (2021). Ms_piano: A software tool for annotating peaks in cid tandem mass spectra of peptides and n-glycopeptides. J. Proteome Res..

[28] Nist ms Search Software. https://chemdata.nist.gov/dokuwiki/doku.php?id=chemdata:downloads:start#nist_ms_search_program.

[29] Remoroza C.A., Burke M.C., Liu Y., Mirokhin Y.A., Tchekhovskoi D.V., Yang X., Stein S.E. (2021). Representing and comparing site-specific glycan abundance distributions of glycoproteins. J. Proteome Res..

[30] R_core_team R: A Language and Environment for Statistical Computing. https://www.R-project.org/.

[31] Letunic I., Bork P. (2024). Interactive tree of life (itol) v6: Recent updates to the phylogenetic tree display and annotation tool. Nucleic Acids Res..

[32] Schoch C.L., Ciufo S., Domrachev M., Hotton C.L., Kannan S., Khovanskaya R., Leipe D., McVeigh R., O’Neill K., Robbertse B. (2020). NCBI taxonomy: A comprehensive update on curation, resources and tools. Database.

[33] McAuley J.L., Gilbertson B.P., Trifkovic S., Brown L.E., McKimm-Breschkin J.L. (2019). Influenza virus neuraminidase structure and functions. Front. Microbiol..

[34] Stein S.E. (1994). Estimating probabilities of correct identification from results of mass spectral library searches. J. Am. Soc. Mass. Spectrom..

[35] Samokhin A., Sotnezova K., Lashin V., Revelsky I. (2015). Evaluation of mass spectral library search algorithms implemented in commercial software. J. Mass. Spectrom..

[36] NIST Mass Spectrometry Data Center (2008). NIST Standard Reference Database 1A: NIST/EPA/NIH Mass Spectral Library (NIST 08) and NIST Mass Spectral Search Program (Version 2.0f), User’s Guide.

[37] Bruce-Staskal P.J., Woods R.M., Borisov O.V., Massare M.J., Hahn T.J. (2020). Hemagglutinin from multiple divergent influenza a and b viruses bind to a distinct branched, sialylated poly-lacnac glycan by surface plasmon resonance. Vaccine.

[38] Burke M.C., Liu Y., Remoroza C., Mirokhin Y.A., Sheetlin S.L., Tchekhovskoi D.V., Wang G., Yang X., Stein S.E. (2023). Determining site-specific glycan profiles of recombinant SARS-CoV-2 spike proteins from multiple sources. J. Proteome Res..

[39] Li J., Liu S., Gao Y., Tian S., Yang Y., Ma N. (2021). Comparison of n-linked glycosylation on hemagglutinins derived from chicken embryos and mdck cells: A case of the production of a trivalent seasonal influenza vaccine. Appl. Microbiol. Biotechnol..

[40] Hackett W.E., Zaia J. (2021). Calculating glycoprotein similarities from mass spectrometric data. Mol. Cell. Proteom..

[41] Remoroza C.A., Burke M.C., Mak T.D., Sheetlin S.L., Mirokhin Y.A., Cooper B.T., Goecker Z.C., Lowenthal M.S., Yang X., Wang G. (2024). Comparison of n-glycopeptide to released n-glycan abundances and the influence of glycopeptide mass and charge states on n-linked glycosylation of igg antibodies. J. Proteome Res..

[42] Li Y., Liu D., Wang Y., Su W., Liu G., Dong W. (2021). The importance of glycans of viral and host proteins in enveloped virus infection. Front. Immunol..

[43] Vulto A.G., Jaquez O.A. (2017). The process defines the product: What really matters in biosimilar design and production?. Rheumatology.

[44] Ampofo W.K., Baylor N., Cobey S., Cox N.J., Daves S., Edwards S., Ferguson N., Grohmann G., Hay A., Katz J. (2012). Improving influenza vaccine virus selection: Report of a who informal consultation held at who headquarters, geneva, switzerland, 14–16 June 2010. Influenza Other Respir. Viruses.

[45] Hong M., Li T., Xue W., Zhang S., Cui L., Wang H., Zhang Y., Zhou L., Gu Y., Xia N. (2022). Genetic engineering of baculovirus-insect cell system to improve protein production. Front. Bioeng. Biotechnol..

[46] Monteiro F., Carinhas N., Carrondo M.J., Bernal V., Alves P.M. (2012). Toward system-level understanding of baculovirus-host cell interactions: From molecular fundamental studies to large-scale proteomics approaches. Front. Microbiol..

[47] U.S. Food and Drug Administration Vaccines Licensed for Use in the United States. https://www.fda.gov/vaccines-blood-biologics/vaccines/vaccines-licensed-use-united-states.

[48] Caval T., Heck A.J.R., Reiding K.R. (2021). Meta-heterogeneity: Evaluating and describing the diversity in glycosylation between sites on the same glycoprotein. Mol. Cell Proteom..

[49] Pritchard L.K., Spencer D.I., Royle L., Bonomelli C., Seabright G.E., Behrens A.J., Kulp D.W., Menis S., Krumm S.A., Dunlop D.C. (2015). Glycan clustering stabilizes the mannose patch of hiv-1 and preserves vulnerability to broadly neutralizing antibodies. Nat. Commun..

[50] Zhu B., Shen J., Zhao T., Jiang H., Ma T., Zhang J., Dang L., Gao N., Hu Y., Shi Y. (2019). Intact glycopeptide analysis of influenza a/h1n1/09 neuraminidase revealing the effects of host and glycosite location on site-specific glycan structures. Proteomics.

[51] Saeidi S., Wan H., Kang H., Gao J., Wu W.W., Malik T., Daniels R. (2025). N-linked glycans on the stalk of influenza virus neuraminidase promote functional tetramer formation by compensating for local hydrophobicity. J. Virol..

[52] de Vries E., Du W., Guo H., de Haan C.A.M. (2020). Influenza a virus hemagglutinin-neuraminidase-receptor balance: Preserving virus motility. Trends Microbiol..

[53] Matsuoka Y., Swayne D.E., Thomas C., Rameix-Welti M.A., Naffakh N., Warnes C., Altholtz M., Donis R., Subbarao K. (2009). Neuraminidase stalk length and additional glycosylation of the hemagglutinin influence the virulence of influenza h5n1 viruses for mice. J. Virol..

[54] Reid C.Q., Tait A., Baldascini H., Mohindra A., Racher A., Bilsborough S., Smales C.M., Hoare M. (2010). Rapid whole monoclonal antibody analysis by mass spectrometry: An ultra scale-down study of the effect of harvesting by centrifugation on the post-translational modification profile. Biotechnol. Bioeng..

[55] Saldova R., Kilcoyne M., Stockmann H., Millan Martin S., Lewis A.M., Tuite C.M., Gerlach J.Q., Le Berre M., Borys M.C., Li Z.J. (2017). Advances in analytical methodologies to guide bioprocess engineering for bio-therapeutics. Methods.

[56] Deutsch E.W., Bandeira N., Perez-Riverol Y., Sharma V., Carver J.J., Mendoza L., Kundu D.J., Wang S., Bandla C., Kamatchinathan S. (2023). The proteomexchange consortium at 10 years: 2023 update. Nucleic Acids Res..

[57] Perez-Riverol Y., Bai J., Bandla C., Garcia-Seisdedos D., Hewapathirana S., Kamatchinathan S., Kundu D.J., Prakash A., Frericks-Zipper A., Eisenacher M. (2022). The pride database resources in 2022: A hub for mass spectrometry-based proteomics evidences. Nucleic Acids Res..

[58] NIST Mass Spectrometry Data Center Protein Subunit Vaccine Glycopeptides. https://chemdata.nist.gov/dokuwiki/doku.php?id=peptidew:protein_subunit_vaccine_glyco.

