# Supplementary material for: Site-Specific Glycosylation Profiling of Protein Subunit and Inactivated Virus Vaccines
Source: Vaccines (Basel). 2026 Jul 22;14(7):644. doi: 10.3390/vaccines14070644 (PMC13417270; doi:10.3390/vaccines14070644)
Supplement: Supplementary file 1 [file vaccines-14-00644-s001.zip › vaccines-4427232-supplementary.pdf]

Supporting Information for:
Site-specific Glycosylation Profiling of Protein Subunit and Inactivated Virus Vaccines

Authors:
Zachary C. Goecker[1]; Meghan C. Burke[1]; Yi Liu[1]; Yuri A. Mirokhin[1]; Sergey L. Sheetlin[1]; Guanghui Wang[1]; Dmitrii V. Tchekhovskoi[1]; Xiaoyu Yang[1]; Stephen E. Stein[1]
[1]Mass Spectrometry Data Center, National Institute of Standards and Technology, 100 Bureau Drive, Gaithersburg, Maryland 20899, United States

SUPPORTING INFORMATION
Page S-1: Table S1. Supplier information
Figure S1. Tables of EPISET identification numbers used to assemble FASTA files
Page S-2: Figure S2. Pairwise comparisons of all good quality GADS
Page S-3: Figure S3. Coverage of detection for all known glycosylation sites in acquired vaccines
Page S-4: Figure S4. GADS Demonstrating a Mixture of Common Classes
Page S-5: Table S2. List of raw files uploaded to repository and corresponding information
Page S-6: Figure S5. Box and Whisker Plot of Replicate Injections and Replicate Digestions
Figure S6. Intra-protein variation of glycosylation distribution within each vaccine
Page S-7: Figure S7. Correlation between glycosylation distribution similarity and site position from different sites on the same protein
Figure S8. Box and Whisker Plot of Intra-protein Variation within Neuraminidase and Hemagglutinin
Page S-8: Figure S9. GADS Comparison of the HA Head Region in Afluria 2022-2023 Formulation
Page S-9: Figure S10. GADS Comparison of the HA Stalk Region in A/NewCaledonia/20/1999
Page S-10: Figure S11. Glycosylation site alignment for hemagglutinin among quadrivalent influenza vaccines
Figure S12. Glycosylation site alignment for neuraminidase among quadrivalent influenza vaccines
Page S-11: Figure S13. Illustration of verification of multiply-fucosylated glycopeptide spectra
Page S-12: Figure S14. GADS similarity as a function of glycosylation site distance
Page S-13: Table S3. Summary of all glycosylation comparisons made in this study at the protein level

Table S1 Supplier Information

Supplier	Identifier
Seqirus	1
NIBSC	2
Creative Biomart	3
Sanofi Pasteur	4
Novavax	5
GlaxoSmithKline	6

Vaccine	EPI_SET IDs
Afluria 2021-2022	EPI1801581
	EPI1801580
	EPI1801578
	EPI1801579
	EPI1801577
	EPI1801574
	EPI1801576
	EPI1801575
	EPI1837753
	EPI1837752
	EPI1841681
	EPI1841682
	EPI1841680
	EPI1841678
	EPI1837751
	EPI1841679
	EPI1629575
	EPI1629574
	EPI1629572
	EPI1629573
	EPI1629571
	EPI1629568
	EPI1629570
	EPI1629569
	EPI1799824
	EPI1799823
	EPI1799821
	EPI1799822
	EPI1799820
	EPI1799817
	EPI1799819
	EPI1799818

Vaccine	EPI_SET IDs
Afluria 2022-2023	EPI1801581
	EPI1801580
	EPI1801578
	EPI1801579
	EPI1801577
	EPI1801574
	EPI1801576
	EPI1801575
	EPI1925255
	EPI1925254
	EPI1925252
	EPI1925253
	EPI1925251
	EPI1925248
	EPI1925250
	EPI1925249
	EPI2413502
	EPI2413500
	EPI2413506
	EPI2413504
	EPI2413501
	EPI2413503
	EPI2413507
	EPI2413505
	EPI1799824
	EPI1799823
	EPI1799821
	EPI1799822
	EPI1799820
	EPI1799817
	EPI1799819
	EPI1799818

Vaccine	EPI_SET IDs
Afluria 2023-2024	EPI2603720
	EPI2603719
	EPI2603717
	EPI2603718
	EPI2603716
	EPI2603713
	EPI2603715
	EPI2603714
	EPI1925255
	EPI1925254
	EPI1925252
	EPI1925253
	EPI1925251
	EPI1925248
	EPI1925250
	EPI1925249
	EPI2413502
	EPI2413500
	EPI2413506
	EPI2413504
	EPI2413501
	EPI2413503
	EPI2413507
	EPI2413505
	EPI1799824
	EPI1799823
	EPI1799821
	EPI1799822
	EPI1799820
	EPI1799817
	EPI1799819
	EPI1799818

Vaccine	EPI_SET IDs
Flublok 2022-2023	EPI1661758
	EPI1661757
	EPI1661755
	EPI1661756
	EPI1661754
	EPI1661751
	EPI1661753
	EPI1661752
	EPI1925255
	EPI1925254
	EPI1925252
	EPI1925253
	EPI1925251
	EPI1925248
	EPI1925250
	EPI1925249
	EPI2413502
	EPI2413500
	EPI2413506
	EPI2413504
	EPI2413501
	EPI2413503
	EPI2413507
	EPI2413505
	EPI1799824
	EPI1799823
	EPI1799821
	EPI1799822
	EPI1799820
	EPI1799817
	EPI1799819
	EPI1799818

Vaccine	EPI_SET IDs
Flucelvax 2022-2023	EPI1705806
	EPI1705805
	EPI1705803
	EPI1705804
	EPI1705802
	EPI1705799
	EPI1705801
	EPI1705800
	EPI1859998
	EPI1859997
	EPI1884986
	EPI1884987
	EPI1884985
	EPI1884982
	EPI1884984
	EPI1884983
	EPI1884735
	EPI1884734
	EPI1916383
	EPI1916384
	EPI1916382
	EPI1916379
	EPI1916381
	EPI1916380
	EPI1051681
	EPI1051680
	EPI1051678
	EPI1051679
	EPI1051677
	EPI1051674
	EPI1051676
	EPI1051675

Vaccine	EPI_SET IDs
Flucelvax 2023-2024	EPI2212483
	EPI2212482
	EPI2212480
	EPI2212481
	EPI2212479
	EPI2212476
	EPI2212478
	EPI2212477
	EPI1859998
	EPI1859997
	EPI1884986
	EPI1884987
	EPI1884985
	EPI1884982
	EPI1884984
	EPI1884983
	EPI1884735
	EPI1884734
	EPI1916383
	EPI1916384
	EPI1916382
	EPI1916379
	EPI1916381
	EPI1916380
	EPI1051681
	EPI1051680
	EPI1051678
	EPI1051679
	EPI1051677
	EPI1051674
	EPI1051676
	EPI1051675

Vaccine	EPI_SET IDs
Fluad 2023-2024	EPI2603720
	EPI2603719
	EPI2603717
	EPI2603718
	EPI2603716
	EPI2603713
	EPI2603715
	EPI2603714
	EPI1925255
	EPI1925254
	EPI1925252
	EPI1925253
	EPI1925251
	EPI1925248
	EPI1925250
	EPI1925249
	EPI2413502
	EPI2413500
	EPI2413506
	EPI2413504
	EPI2413501
	EPI2413503
	EPI2413507
	EPI2413505
	EPI1799824
	EPI1799823
	EPI1799821
	EPI1799822
	EPI1799820
	EPI1799817
	EPI1799819
	EPI1799818

Vaccine	EPI_SET IDs
A/New Caledonia/20/1999 (H1N1)	ACF41878.1
	ACF41881.1
	ACF41883.1
	ACF41884.1
	ACF41879.1
	ADL41185.1
	ACX46209.1
	ABV01075.1
	ACF41888.1
	ACF41885.1

Vaccine	EPI_SET IDs
A/Panama/2007/1999 (H3N2)	EPI362901
	EPI362903
	EPI362904
	EPI362906
	EPI362907
	EPI362905
	EPI362902
	EPI362902
	EPI137439

Vaccine	EPI_SET IDs
A/Philippines/2/1982 (H3N2)	ADJ41805.1
	ADJ41808.1
	ADJ41810.1
	ADJ41811.1
	ADJ41806.1
	ADJ41807.1
	ADJ41809.1
	ADJ41813.1
	ADJ41815.1
	ADJ41812.1

Vaccine	EPI_SET IDs
A/Shandong/9/1993 (H3N2)	EPI390010
	EPI239754
	EPI390016
	EPI390017
	EPI390015
	EPI390013
	EPI390011
	EPI390014

Vaccine	EPI_SET IDs
A/Switzerland/9715293/2013 (H3N2)	EPI540526
	EPI540525
	EPI540520
	EPI540521
	EPI540519
	EPI540524
	EPI540523
	EPI540522

Figure S1. **Tables of EPISET identification numbers used to assemble FASTA files**

Protein sequences for peptide identification were obtained from the Global Initiative on Sharing All Influenza Data (GISAID.org). Protein sequences are not directly shared in this work, but may be accessed by searching for EPISET IDs within EpiFlu.

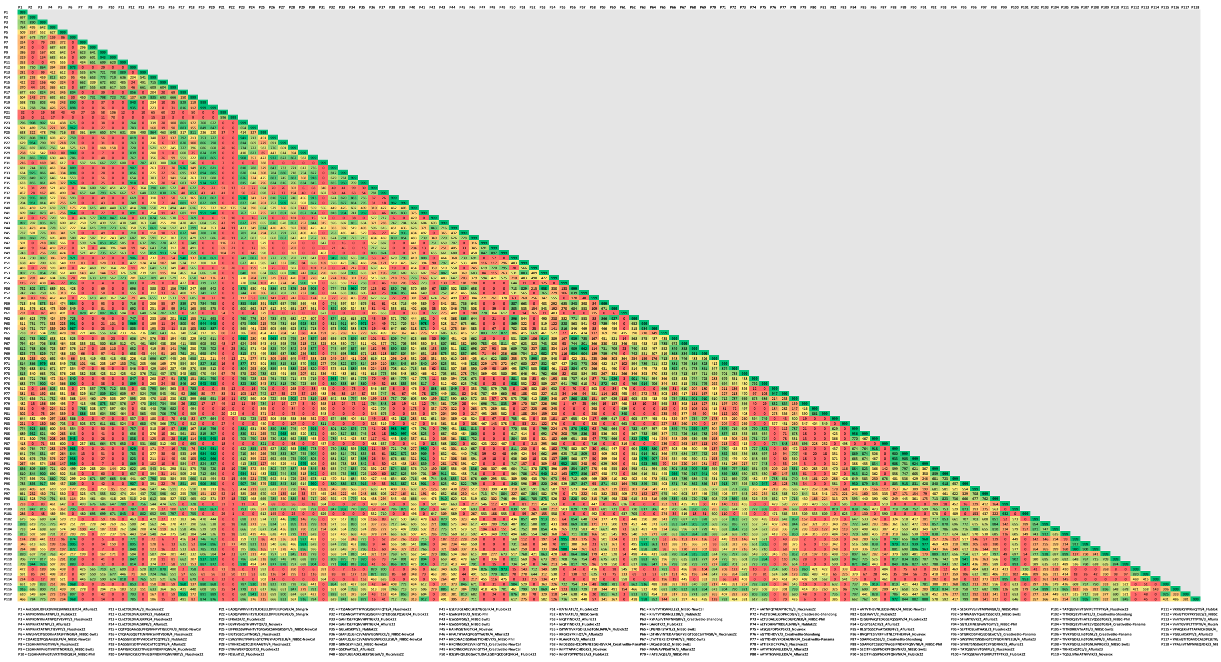

Figure S2. **Pairwise comparisons of all good quality GADS**
All GADS from influenza vaccines with numbers of identified glycopeptides (nSpec > 40) were compared, excluding replicates. These scores represent dot product. Note that a 0 indicates no peaks in common, whereas a score of 999 indicates identical spectra. A threshold similarity score of 300 was used for clustering into groups. Six such GADS classes resulted. These classes are employed in Figure 2 of the manuscript.

Vaccine	Glycoprotein	Strains	Glycosylation sites
Afluria Quadrivalent 2021	Hemagglutinin	A/Victoria/2570/2019	27 28 40 104 179 293 304 498 557
		A/Cambodia/e0826360/2020	24 38 54 61 79 138 142 149 174 181 262 301 499
		B/Victoria/705/2018	40 74 160 178 245 316 345 504 530 543
		B/Phuket/3073/2013	40 74 160 182 211 318 347 506 532 545 577
Afluria Quadrivalent 2022	Hemagglutinin	A/Victoria/2570/2019	27 28 40 104 179 293 304 498 557
		A/Darwin/6/2021	24 38 54 61 79 138 142 149 181 262 301 499
		B/Austria/1359417/2021	40 74 160 178 245 316 345 504 530 543 575
		B/Phuket/3073/2013	40 74 160 182 211 318 347 506 532 545 577
Afluria Quadrivalent 2023	Hemagglutinin	A/Victoria/4897/2022	27 28 40 104 179 293 304 498 557
		A/Darwin/6/2021	24 38 54 61 79 138 142 149 181 262 301 499
		B/Austria/1359417/2021	40 74 160 178 245 316 345 504 530 543 575
		B/Phuket/3073/2013	40 74 160 182 211 318 347 506 532 545 577
Flublok Quadrivalent 2022	Hemagglutinin	A/Wisconsin/588/2019	27 28 40 104 179 293 304 498 557
		A/Darwin/6/2021	24 38 54 61 79 138 142 149 181 262 301 499
		B/Austria/1359417/2021	40 74 160 178 245 316 345 504 530 543 575
		B/Phuket/3073/2013	40 74 160 182 211 318 347 506 532 545 577
Flucelvax Quadrivalent 2022	Hemagglutinin	A/Delaware/55/2019	27 28 40 104 179 293 304 498 557
		A/Darwin/11/2021	24 38 54 61 79 138 142 149 181 301 499
		B/Singapore/WUH4618/2021	40 74 160 178 245 316 345 504 530 543 575
		B/Singapore/INFTT-16-0610/2016	40 74 160 182 318 347 506 532 545 577
Flucelvax Quadrivalent 2023	Hemagglutinin	A/Georgia/12/2022	27 28 40 104 179 293 304 498 557
		A/Darwin/11/2021	24 38 54 61 79 138 142 149 181 301 499
		B/Singapore/WUH4618/2021	40 74 160 178 245 316 345 504 530 543 575
		B/Singapore/INFTT-16-0610/2016	40 74 160 182 318 347 506 532 545 577
Fluad Quadrivalent 2023	Hemagglutinin	A/Victoria/4897/2022	27 28 40 104 179 293 304 498 557
		A/Darwin/6/2021	24 38 54 61 79 138 142 149 181 262 301 499
		B/Austria/1359417/2021	40 74 160 178 245 316 345 504 530 543 575
		B/Phuket/3073/2013	40 74 160 182 211 318 347 506 532 545 577
Afluria Quadrivalent 2021	Neuraminidase	A/Victoria/2570/2019	42 50 58 63 68 88 146 235
		A/Cambodia/e0826360/2020	61 70 86 146 200 234 245 367 463
		B/Victoria/705/2018	56 64 144 284
		B/Phuket/3073/2013	56 64 144 284 463
Afluria Quadrivalent 2022	Neuraminidase	A/Victoria/2570/2019	42 50 58 63 68 88 146 235
		A/Darwin/6/2021	61 70 86 146 200 234 245 367 463
		B/Austria/1359417/2021	56 64 144 284
		B/Phuket/3073/2013	56 64 144 284 463
Afluria Quadrivalent 2023	Neuraminidase	A/Victoria/4897/2022	42 58 63 68 88 146 235
		A/Darwin/6/2021	61 70 86 146 200 234 245 367 463
		B/Austria/1359417/2021	56 64 144 284
		B/Phuket/3073/2013	56 64 144 284 463
Flublok Quadrivalent 2022	Neuraminidase	A/Wisconsin/588/2019	42 50 58 63 68 88 146 235
		A/Darwin/6/2021	61 70 86 146 200 234 245 367 463
		B/Austria/1359417/2021	56 64 144 284
		B/Phuket/3073/2013	56 64 144 284 463
Flucelvax Quadrivalent 2022	Neuraminidase	A/Delaware/55/2019	42 50 58 63 68 88 146 235
		A/Darwin/11/2021	61 70 86 146 200 234 245 367 463
		B/Singapore/WUH4618/2021	56 64 145 285
		B/Singapore/INFTT-16-0610/2016	56 64 144 284 463
Flucelvax Quadrivalent 2023	Neuraminidase	A/Georgia/12/2022	42 50 58 63 68 88 146 235
		A/Darwin/11/2021	61 70 86 146 200 234 245 367 463
		B/Singapore/WUH4618/2021	56 64 145 285
		B/Singapore/INFTT-16-0610/2016	56 64 144 284 463
Fluad Quadrivalent 2023	Neuraminidase	A/Victoria/4897/2022	42 58 63 68 88 146 235
		A/Darwin/6/2021	61 70 86 146 200 234 245 367 463
		B/Austria/1359417/2021	56 64 144 284
		B/Phuket/3073/2013	56 64 144 284 463
NIBSC Influenza A	Hemagglutinin	A/NewCaledonia/20/1999	27 28 40 71 104 142 176 303 497 556
NIBSC Influenza A	Hemagglutinin	A/Philippines/2/1982	24 38 54 79 142 160 181 262 301 499
NIBSC Influenza A	Hemagglutinin	A/Switzerland/9715293/2013	24 38 54 61 79 138 149 160 181 262 301 499
Creative Biomart Influenza A	Hemagglutinin	A/Panama/2007/1999	24 38 54 79 138 142 149 160 181 262 301 499
Creative Biomart Influenza A	Hemagglutinin	A/NewCaledonia/20/1999	27 28 40 71 104 142 176 303 497 556
Creative Biomart Influenza A	Hemagglutinin	A/Shandong/9/1993	24 38 54 79 142 181 262 292 301 499
NIBSC Influenza A	Neuraminidase	A/NewCaledonia/20/1999	44 58 63 70 88 146 235 434 455
NIBSC Influenza A	Neuraminidase	A/Philippines/2/1982	61 70 86 146 200 234 402
NIBSC Influenza A	Neuraminidase	A/Switzerland/9715293/2013	61 70 86 146 200 234 329 367
Creative Biomart Influenza A	Neuraminidase	A/Panama/2007/1999	61 70 86 146 200 234 329 402
Creative Biomart Influenza A	Neuraminidase	A/NewCaledonia/20/1999	44 58 63 70 88 146 235 434 455
Creative Biomart Influenza A	Neuraminidase	A/Shandong/9/1993	61 70 86 146 200 234 329 402
Novavax Covid-19 2023	Spike Protein	Omicron XBB.1.5	58 71 119 145 161 230 278 327 339 599 612 653 709 713 797 1070 1094 1130 1154 1169 1190
Shingrix	Surface Glycoprotein E		266 437 577

Figure S3. **Coverage of detection for all known glycosylation sites in acquired vaccines**
All known glycosylation sites are listed for glycoproteins of interest. Sites that are not highlighted indicate detected glycosylation distributions. Sites highlighted in red indicate a lack of detection either due to insufficient signal or indistinguishable sequences with other strains in the sample.

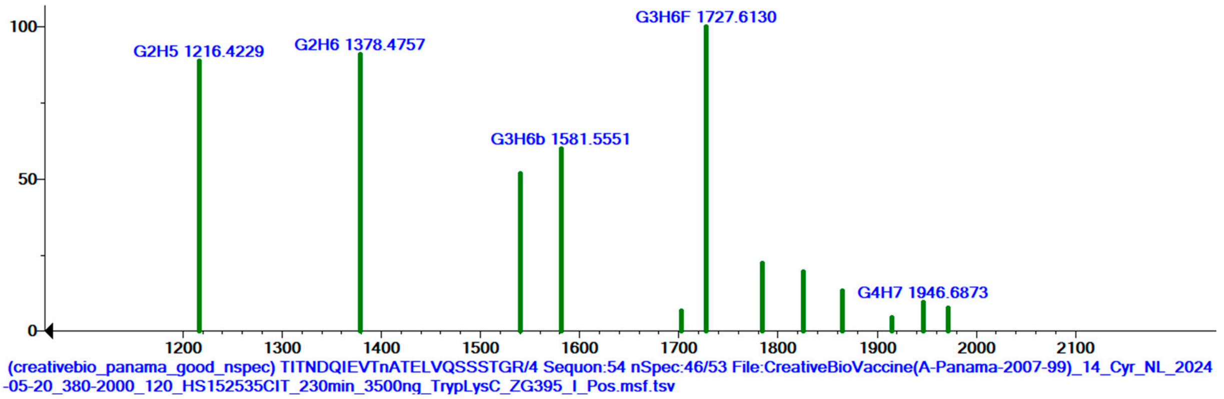

Figure S4. **GADS Demonstrating a Mixture of Common Classes**
This is a depiction of a glycosylation distribution which is a mixture of GADS classes as it contains high-mannose glycans, hybrid glycans, and complex glycans. This distribution was taken from sequon 54 of HA of strain A/Panama/2007/1999 produced by supplier 3.

Table S2. **List of raw files uploaded to repository and corresponding information**

File name	Vaccine	Sample Identifier	Source	Notes
Afluria-vaccine_08_RG_NL_2022-06-03_380-2000_120_HS152535CIT_230min_2ug_TrypGluC_ZG183_I_Pos.raw	Afluria 2021-2022	ZG183	Native in egg	
Afluria-vaccine_10_RG_NL_2022-06-03_380-2000_120_HS152535CIT_230min_2ug_ChymoGluC_ZG184_I_Pos.raw	Afluria 2021-2022	ZG184	Native in egg	
Afluria-vaccine_11_RG_NL_2022-06-03_380-2000_120_HS152535CIT_230min_2ug_TrypChymo_ZG185_I_Pos.raw	Afluria 2021-2022	ZG185	Native in egg	
Afluria-vaccine_12_RG_NL_2022-06-03_380-2000_120_HS152535CIT_230min_2ug_Chymo_ZG186_I_Pos.raw	Afluria 2021-2022	ZG186	Native in egg	
Afluria-vaccine_13_RG_NL_2022-06-03_380-2000_120_HS152535CIT_230min_2ug_alPWT_ZG187_I_Pos.raw	Afluria 2021-2022	ZG187	Native in egg	
Afluria-vaccine_14_RG_NL_2022-06-03_380-2000_120_HS152535CIT_230min_2ug_TrypLysC_ZG188_I_Pos.raw	Afluria 2021-2022	ZG188	Native in egg	
Afluria-vaccine_06_NL_2022-07-18_380-2000_120_HS152535CIT_230min_2ug_TrypGluC_ZG183-rerun_I_Pos.raw	Afluria 2021-2022	ZG183	Native in egg	Injection replicate
Afluria-vaccine_07_NL_2022-07-18_380-2000_120_HS152535CIT_230min_2ug_ChymoGluC_ZG184-rerun_I_Pos.raw	Afluria 2021-2022	ZG184	Native in egg	Injection replicate
Afluria-vaccine_08_NL_2022-07-18_380-2000_120_HS152535CIT_230min_2ug_TrypChymo_ZG185-rerun_I_Pos.raw	Afluria 2021-2022	ZG185	Native in egg	Injection replicate
Afluria-vaccine_01_Dial-RG_NL_2022-07-25_380-2000_120_HS152535CIT_230min_2ug_Chymo_ZG186-rerun_I_Pos.raw	Afluria 2021-2022	ZG186	Native in egg	Injection replicate
Afluria-vaccine_02_Dial-RG_NL_2022-07-25_380-2000_120_HS152535CIT_230min_2ug_alPWT_ZG187-rerun_I_Pos.raw	Afluria 2021-2022	ZG187	Native in egg	Injection replicate
Afluria-vaccine_03_Dial-RG_NL_2022-07-25_380-2000_120_HS152535CIT_230min_2ug_TrypLysC_ZG188-rerun_I_Pos.raw	Afluria 2021-2022	ZG188	Native in egg	Injection replicate
Afluria2021_01_Cyr_NL_2023-08-31_380-2000_120_HS152535CIT_230min_2ug_TrypGluC_ZG358_I_Pos.raw	Afluria 2021-2022	ZG358	Native in egg	Digestion replicate
Afluria2021_02_Cyr_NL_2023-08-31_380-2000_120_HS152535CIT_230min_2ug_TrypLysC_ZG359_I_Pos.raw	Afluria 2021-2022	ZG358	Native in egg	Digestion replicate
Afluria2021_03_Cyr_NL_2023-08-31_380-2000_120_HS152535CIT_230min_2ug_TrypChymo_ZG360_I_Pos.raw	Afluria 2021-2022	ZG358	Native in egg	Digestion replicate
Afluria2021_04_Cyr_NL_2023-08-31_380-2000_120_HS152535CIT_230min_2ug_ChymoGluC_ZG361_I_Pos.raw	Afluria 2021-2022	ZG358	Native in egg	Digestion replicate
Afluria2021_05_Cyr_NL_2023-08-31_380-2000_120_HS152535CIT_230min_2ug_Chymo_ZG362_I_Pos.raw	Afluria 2021-2022	ZG358	Native in egg	Digestion replicate
Afluria2021_06_Cyr_NL_2023-08-31_380-2000_120_HS152535CIT_230min_2ug_alPWT_ZG363_I_Pos.raw	Afluria 2021-2022	ZG358	Native in egg	Digestion replicate
Afluria22-23_02_Cyr_NL_2024-07-18_380-2000_120_HS152535CIT_230min_2ug_TrypGluC_ZG446_I_Pos.raw	Afluria 2022-2023	ZG446	Native in egg	
Afluria22-23_03_Cyr_NL_2024-07-18_380-2000_120_HS152535CIT_230min_2ug_TrypLysC_ZG447_I_Pos.raw	Afluria 2022-2023	ZG447	Native in egg	
Afluria22-23_04_Cyr_NL_2024-07-18_380-2000_120_HS152535CIT_230min_2ug_TrypChymo_ZG448_I_Pos.raw	Afluria 2022-2023	ZG448	Native in egg	
Afluria22-23_05_Cyr_NL_2024-07-18_380-2000_120_HS152535CIT_230min_2ug_ChymoGluC_ZG449_I_Pos.raw	Afluria 2022-2023	ZG449	Native in egg	
Afluria22-23_06_Cyr_NL_2024-07-18_380-2000_120_HS152535CIT_230min_2ug_Chymo_ZG450_I_Pos.raw	Afluria 2022-2023	ZG450	Native in egg	
Afluria22-23_07_Cyr_NL_2024-07-18_380-2000_120_HS152535CIT_230min_2ug_alPWT_ZG451_I_Pos.raw	Afluria 2022-2023	ZG451	Native in egg	
Afluria22-23_08_Cyr_NL_2024-07-18_380-2000_120_HS152535CIT_230min_2ug_TrypGluC_ZG446_I_Pos.raw	Afluria 2022-2023	ZG446	Native in egg	Injection replicate
Afluria22-23_09_Cyr_NL_2024-07-18_380-2000_120_HS152535CIT_230min_2ug_TrypLysC_ZG447_I_Pos.raw	Afluria 2022-2023	ZG447	Native in egg	Injection replicate
Afluria22-23_10_Cyr_NL_2024-07-18_380-2000_120_HS152535CIT_230min_2ug_TrypChymo_ZG448_I_Pos.raw	Afluria 2022-2023	ZG448	Native in egg	Injection replicate
Afluria22-23_11_Cyr_NL_2024-07-18_380-2000_120_HS152535CIT_230min_2ug_ChymoGluC_ZG449_I_Pos.raw	Afluria 2022-2023	ZG449	Native in egg	Injection replicate
Afluria22-23_12_Cyr_NL_2024-07-18_380-2000_120_HS152535CIT_230min_2ug_Chymo_ZG450_I_Pos.raw	Afluria 2022-2023	ZG450	Native in egg	Injection replicate
Afluria22-23_13_Cyr_NL_2024-07-18_380-2000_120_HS152535CIT_230min_2ug_alPWT_ZG451_I_Pos.raw	Afluria 2022-2023	ZG451	Native in egg	Injection replicate
Afluria22-23_01_Cyr_NL_2023-02-17_380-2000_120_HS152535CIT_230min_2ug_TrypGluC_ZG308_I_Pos.raw	Afluria 2022-2023	ZG308	Native in egg	Digestion replicate
Afluria22-23_02_Cyr_NL_2023-02-17_380-2000_120_HS152535CIT_230min_2ug_TrypLysC_ZG309_I_Pos.raw	Afluria 2022-2023	ZG309	Native in egg	Digestion replicate
Afluria22-23_03_Cyr_NL_2023-02-17_380-2000_120_HS152535CIT_230min_2ug_TrypChymo_ZG310_I_Pos.raw	Afluria 2022-2023	ZG310	Native in egg	Digestion replicate
Afluria22-23_04_Cyr_NL_2023-02-17_380-2000_120_HS152535CIT_230min_2ug_ChymoGluC_ZG299_I_Pos.raw	Afluria 2022-2023	ZG299	Native in egg	Digestion replicate
Afluria22-23_05_Cyr_NL_2023-02-17_380-2000_120_HS152535CIT_230min_2ug_Chymo_ZG300_I_Pos.raw	Afluria 2022-2023	ZG300	Native in egg	Digestion replicate
Afluria22-23_06_Cyr_NL_2023-02-17_380-2000_120_HS152535CIT_230min_2ug_alPWT_ZG301_I_Pos.raw	Afluria 2022-2023	ZG301	Native in egg	Digestion replicate
Afluria23-24_07_Cyr_NL_2024-08-15_380-2000_120_HS152535CIT_230min_2ug_TrypGluC_ZG470_I_Pos.raw	Afluria 2023-2024	ZG470	Native in egg	
Afluria23-24_08_Cyr_NL_2024-08-15_380-2000_120_HS152535CIT_230min_2ug_TrypLysC_ZG471_I_Pos.raw	Afluria 2023-2024	ZG471	Native in egg	
Afluria23-24_09_Cyr_NL_2024-08-15_380-2000_120_HS152535CIT_230min_2ug_TrypChymo_ZG472_I_Pos.raw	Afluria 2023-2024	ZG472	Native in egg	
Afluria23-24_10_Cyr_NL_2024-08-15_380-2000_120_HS152535CIT_230min_2ug_ChymoGluC_ZG473_I_Pos.raw	Afluria 2023-2024	ZG473	Native in egg	
Afluria23-24_11_Cyr_NL_2024-08-15_380-2000_120_HS152535CIT_230min_2ug_Chymo_ZG474_I_Pos.raw	Afluria 2023-2024	ZG474	Native in egg	
Afluria23-24_12_Cyr_NL_2024-08-15_380-2000_120_HS152535CIT_230min_2ug_alPWT_ZG475_I_Pos.raw	Afluria 2023-2024	ZG475	Native in egg	
Afluria23-24_13_Cyr_NL_2024-08-15_380-2000_120_HS152535CIT_230min_2ug_TrypGluC_ZG470_I_Pos.raw	Afluria 2023-2024	ZG470	Native in egg	Injection replicate
Afluria23-24_14_Cyr_NL_2024-08-15_380-2000_120_HS152535CIT_230min_2ug_TrypLysC_ZG471_I_Pos.raw	Afluria 2023-2024	ZG471	Native in egg	Injection replicate
Afluria23-24_15_Cyr_NL_2024-08-15_380-2000_120_HS152535CIT_230min_2ug_TrypChymo_ZG472_I_Pos.raw	Afluria 2023-2024	ZG472	Native in egg	Injection replicate
Afluria23-24_16_Cyr_NL_2024-08-15_380-2000_120_HS152535CIT_230min_2ug_ChymoGluC_ZG473_I_Pos.raw	Afluria 2023-2024	ZG473	Native in egg	Injection replicate
Afluria23-24_17_Cyr_NL_2024-08-15_380-2000_120_HS152535CIT_230min_2ug_Chymo_ZG474_I_Pos.raw	Afluria 2023-2024	ZG474	Native in egg	Injection replicate
Afluria23-24_18_Cyr_NL_2024-08-15_380-2000_120_HS152535CIT_230min_2ug_alPWT_ZG475_I_Pos.raw	Afluria 2023-2024	ZG475	Native in egg	Injection replicate
Afluria23-24_01_Cyr_NL_2024-08-15_380-2000_120_HS152535CIT_230min_2ug_TrypGluC_ZG464_I_Pos.raw	Afluria 2023-2024	ZG464	Native in egg	Digestion replicate
Afluria23-24_02_Cyr_NL_2024-08-15_380-2000_120_HS152535CIT_230min_2ug_TrypLysC_ZG465_I_Pos.raw	Afluria 2023-2024	ZG465	Native in egg	Digestion replicate
Afluria23-24_03_Cyr_NL_2024-08-15_380-2000_120_HS152535CIT_230min_2ug_TrypChymo_ZG466_I_Pos.raw	Afluria 2023-2024	ZG466	Native in egg	Digestion replicate
Afluria23-24_04_Cyr_NL_2024-08-15_380-2000_120_HS152535CIT_230min_2ug_ChymoGluC_ZG467_I_Pos.raw	Afluria 2023-2024	ZG467	Native in egg	Digestion replicate
Afluria23-24_05_Cyr_NL_2024-08-15_380-2000_120_HS152535CIT_230min_2ug_Chymo_ZG468_I_Pos.raw	Afluria 2023-2024	ZG468	Native in egg	Digestion replicate
Afluria23-24_06_Cyr_NL_2024-08-15_380-2000_120_HS152535CIT_230min_2ug_alPWT_ZG469_I_Pos.raw	Afluria 2023-2024	ZG469	Native in egg	Digestion replicate
Flucelvax22-23_01_In-Gel_NL_2024-09-18_380-2000_120_HS152535CIT_230min_1250ng_TrypGluC_ZG492_I_Pos.raw	Flucelvax 2022-2023	ZG492	Native in MDCK cell culture	
Flucelvax22-23_02_In-Gel_NL_2024-09-18_380-2000_120_HS152535CIT_230min_1250ng_TrypLysC_ZG493_I_Pos.raw	Flucelvax 2022-2023	ZG493	Native in MDCK cell culture	
Flucelvax22-23_01_In-Gel_NL_2024-10-08_380-2000_120_HS152535CIT_230min_3750ng_TrypChymo_ZG498_I_Pos.raw	Flucelvax 2022-2023	ZG498	Native in MDCK cell culture	
Flucelvax22-23_02_In-Gel_NL_2024-10-08_380-2000_120_HS152535CIT_230min_3750ng_ChymoGluC_ZG499_I_Pos.raw	Flucelvax 2022-2023	ZG499	Native in MDCK cell culture	
Flucelvax22-23_03_In-Gel_NL_2024-10-08_380-2000_120_HS152535CIT_230min_3750ng_Chymo_ZG500_I_Pos.raw	Flucelvax 2022-2023	ZG500	Native in MDCK cell culture	
Flucelvax22-23_04_In-Gel_NL_2024-10-08_380-2000_120_HS152535CIT_230min_3750ng_alPWT_ZG501_I_Pos.raw	Flucelvax 2022-2023	ZG501	Native in MDCK cell culture	
Flucelvax22-23_07_In-Gel_NL_2024-09-18_380-2000_120_HS152535CIT_230min_1250ng_TrypGluC_ZG492_I_Pos.raw	Flucelvax 2022-2023	ZG492	Native in MDCK cell culture	Injection replicate
Flucelvax22-23_08_In-Gel_NL_2024-09-18_380-2000_120_HS152535CIT_230min_1250ng_TrypLysC_ZG493_I_Pos.raw	Flucelvax 2022-2023	ZG493	Native in MDCK cell culture	Injection replicate
Flucelvax22-23_05_In-Gel_NL_2024-10-08_380-2000_120_HS152535CIT_230min_3750ng_TrypChymo_ZG498_I_Pos.raw	Flucelvax 2022-2023	ZG498	Native in MDCK cell culture	Injection replicate
Flucelvax22-23_06_In-Gel_NL_2024-10-08_380-2000_120_HS152535CIT_230min_3750ng_ChymoGluC_ZG499_I_Pos.raw	Flucelvax 2022-2023	ZG499	Native in MDCK cell culture	Injection replicate
Flucelvax22-23_07_In-Gel_NL_2024-10-08_380-2000_120_HS152535CIT_230min_3750ng_Chymo_ZG500_I_Pos.raw	Flucelvax 2022-2023	ZG500	Native in MDCK cell culture	Injection replicate
Flucelvax22-23_08_In-Gel_NL_2024-10-08_380-2000_120_HS152535CIT_230min_3750ng_alPWT_ZG501_I_Pos.raw	Flucelvax 2022-2023	ZG501	Native in MDCK cell culture	Injection replicate
Flucelvax22-23_09_In-Gel_NL_2024-10-08_380-2000_120_HS152535CIT_230min_3750ng_TrypGluC_ZG502_I_Pos.raw	Flucelvax 2022-2023	ZG502	Native in MDCK cell culture	Digestion replicate
Flucelvax22-23_10_In-Gel_NL_2024-10-08_380-2000_120_HS152535CIT_230min_3750ng_TrypLysC_ZG503_I_Pos.raw	Flucelvax 2022-2023	ZG503	Native in MDCK cell culture	Digestion replicate
Flucelvax22-23_11_In-Gel_NL_2024-10-08_380-2000_120_HS152535CIT_230min_3750ng_TrypChymo_ZG504_I_Pos.raw	Flucelvax 2022-2023	ZG504	Native in MDCK cell culture	Digestion replicate
Flucelvax22-23_12_In-Gel_NL_2024-10-08_380-2000_120_HS152535CIT_230min_3750ng_ChymoGluC_ZG505_I_Pos.raw	Flucelvax 2022-2023	ZG505	Native in MDCK cell culture	Digestion replicate
Flucelvax22-23_13_In-Gel_NL_2024-10-08_380-2000_120_HS152535CIT_230min_3750ng_Chymo_ZG506_I_Pos.raw	Flucelvax 2022-2023	ZG506	Native in MDCK cell culture	Digestion replicate
Flucelvax22-23_14_In-Gel_NL_2024-10-08_380-2000_120_HS152535CIT_230min_3750ng_alPWT_ZG507_I_Pos.raw	Flucelvax 2022-2023	ZG507	Native in MDCK cell culture	Digestion replicate
Flucelvax22-23_07_Cyr_NL_2023-02-17_380-2000_120_HS152535CIT_230min_2ug_TrypGluC_ZG302_I_Pos.raw	Flucelvax 2022-2023	ZG302	Native in MDCK cell culture	In-solution digestion
Flucelvax22-23_08_Cyr_NL_2023-02-17_380-2000_120_HS152535CIT_230min_2ug_TrypLysC_ZG303_I_Pos.raw	Flucelvax 2022-2023	ZG303	Native in MDCK cell culture	In-solution digestion
Flucelvax22-23_09_Cyr_NL_2023-02-17_380-2000_120_HS152535CIT_230min_2ug_TrypChymo_ZG304_I_Pos.raw	Flucelvax 2022-2023	ZG304	Native in MDCK cell culture	In-solution digestion
Flucelvax22-23_10_Cyr_NL_2023-02-17_380-2000_120_HS152535CIT_230min_2ug_ChymoGluC_ZG305_I_Pos.raw	Flucelvax 2022-2023	ZG305	Native in MDCK cell culture	In-solution digestion
Flucelvax22-23_11_Cyr_NL_2023-02-17_380-2000_120_HS152535CIT_230min_2ug_Chymo_ZG306_I_Pos.raw	Flucelvax 2022-2023	ZG306	Native in MDCK cell culture	In-solution digestion
Flucelvax22-23_12_Cyr_NL_2023-02-17_380-2000_120_HS152535CIT_230min_2ug_alPWT_ZG307_I_Pos.raw	Flucelvax 2022-2023	ZG307	Native in MDCK cell culture	In-solution digestion
Flucelvax23-24_07_Amicon-Cyr_NL_2024-05-06_380-2000_120_HS152535CIT_230min_2ug_TrypGluC_ZG440_I_Pos.raw	Flucelvax 2023-2024	ZG440	Native in MDCK cell culture	
Flucelvax23-24_08_Amicon-Cyr_NL_2024-05-06_380-2000_120_HS152535CIT_230min_2ug_TrypLysC_ZG441_I_Pos.raw	Flucelvax 2023-2024	ZG441	Native in MDCK cell culture	
Flucelvax23-24_09_Amicon-Cyr_NL_2024-05-06_380-2000_120_HS152535CIT_230min_2ug_TrypChymo_ZG442_I_Pos.raw	Flucelvax 2023-2024	ZG442	Native in MDCK cell culture	
Flucelvax23-24_10_Amicon-Cyr_NL_2024-05-06_380-2000_120_HS152535CIT_230min_2ug_ChymoGluC_ZG443_I_Pos.raw	Flucelvax 2023-2024	ZG443	Native in MDCK cell culture	
Flucelvax23-24_11_Amicon-Cyr_NL_2024-05-06_380-2000_120_HS152535CIT_230min_2ug_Chymo_ZG444_I_Pos.raw	Flucelvax 2023-2024	ZG444	Native in MDCK cell culture	
Flucelvax23-24_12_Amicon-Cyr_NL_2024-05-06_380-2000_120_HS152535CIT_230min_2ug_alPWT_ZG445_I_Pos.raw	Flucelvax 2023-2024	ZG445	Native in MDCK cell culture	
Flucelvax23-24_13_Amicon-Cyr_NL_2024-05-06_380-2000_120_HS152535CIT_230min_2ug_TrypGluC_ZG440_I_Pos.raw	Flucelvax 2023-2024	ZG440	Native in MDCK cell culture	Injection replicate
Flucelvax23-24_14_Amicon-Cyr_NL_2024-05-06_380-2000_120_HS152535CIT_230min_2ug_TrypLysC_ZG441_I_Pos.raw	Flucelvax 2023-2024	ZG441	Native in MDCK cell culture	Injection replicate
Flucelvax23-24_15_Amicon-Cyr_NL_2024-05-06_380-2000_120_HS152535CIT_230min_2ug_TrypChymo_ZG442_I_Pos.raw	Flucelvax 2023-2024	ZG442	Native in MDCK cell culture	Injection replicate
Flucelvax23-24_16_Amicon-Cyr_NL_2024-05-06_380-2000_120_HS152535CIT_230min_2ug_ChymoGluC_ZG443_I_Pos.raw	Flucelvax 2023-2024	ZG443	Native in MDCK cell culture	Injection replicate
Flucelvax23-24_17_Amicon-Cyr_NL_2024-05-06_380-2000_120_HS152535CIT_230min_2ug_Chymo_ZG444_I_Pos.raw	Flucelvax 2023-2024	ZG444	Native in MDCK cell culture	Injection replicate
Flucelvax23-24_18_Amicon-Cyr_NL_2024-05-06_380-2000_120_HS152535CIT_230min_2ug_alPWT_ZG445_I_Pos.raw	Flucelvax 2023-2024	ZG445	Native in MDCK cell culture	Injection replicate
Flucelvax23-24_01_Amicon-Cyr_NL_2024-05-06_380-2000_120_HS152535CIT_230min_2ug_TrypGluC_ZG434_I_Pos.raw	Flucelvax 2023-2024	ZG434	Native in MDCK cell culture	Digestion replicate
Flucelvax23-24_02_Amicon-Cyr_NL_2024-05-06_380-2000_120_HS152535CIT_230min_2ug_TrypLysC_ZG435_I_Pos.raw	Flucelvax 2023-2024	ZG435	Native in MDCK cell culture	Digestion replicate
Flucelvax23-24_03_Amicon-Cyr_NL_2024-05-06_380-2000_120_HS152535CIT_230min_2ug_TrypChymo_ZG436_I_Pos.raw	Flucelvax 2023-2024	ZG436	Native in MDCK cell culture	Digestion replicate
Flucelvax23-24_04_Amicon-Cyr_NL_2024-05-06_380-2000_120_HS152535CIT_230min_2ug_ChymoGluC_ZG437_I_Pos.raw	Flucelvax 2023-2024	ZG437	Native in MDCK cell culture	Digestion replicate
Flucelvax23-24_05_Amicon-Cyr_NL_2024-05-06_380-2000_120_HS152535CIT_230min_2ug_Chymo_ZG438_I_Pos.raw	Flucelvax 2023-2024	ZG438	Native in MDCK cell culture	Digestion replicate
Flucelvax23-24_06_Amicon-Cyr_NL_2024-05-06_380-2000_120_HS152535CIT_230min_2ug_alPWT_ZG439_I_Pos.raw	Flucelvax 2023-2024	ZG439	Native in MDCK cell culture	Digestion replicate
Flublok22-23_03_In-Gel_NL_2024-09-18_380-2000_120_HS152535CIT_230min_1250ng_TrypGluC_ZG494_I_Pos.raw	Flublok 2022-2023	ZG494	SF9 recombinant	
Flublok22-23_04_In-Gel_NL_2024-09-18_380-2000_120_HS152535CIT_230min_1250ng_TrypLysC_ZG495_I_Pos.raw	Flublok 2022-2023	ZG495	SF9 recombinant	
Flublok22-23_01_In-Gel_NL_2024-10-25_380-2000_120_HS152535CIT_230min_3750ng_TrypChymo_ZG508_I_Pos.raw	Flublok 2022-2023	ZG508	SF9 recombinant	
Flublok22-23_02_In-Gel_NL_2024-10-25_380-2000_120_HS152535CIT_230min_3750ng_ChymoGluC_ZG509_I_Pos.raw	Flublok 2022-2023	ZG509	SF9 recombinant	
Flublok22-23_03_In-Gel_NL_2024-10-25_380-2000_120_HS152535CIT_230min_3750ng_Chymo_ZG510_I_Pos.raw	Flublok 2022-2023	ZG510	SF9 recombinant	
Flublok22-23_04_In-Gel_NL_2024-10-25_380-2000_120_HS152535CIT_230min_3750ng_alPWT_ZG511_I_Pos.raw	Flublok 2022-2023	ZG511	SF9 recombinant	
Flublok22-23_09_In-Gel_NL_2024-09-18_380-2000_120_HS152535CIT_230min_1250ng_TrypGluC_ZG494_I_Pos.raw	Flublok 2022-2023	ZG494	SF9 recombinant	Injection replicate
Flublok22-23_10_In-Gel_NL_2024-09-18_380-2000_120_HS152535CIT_230min_1250ng_TrypLysC_ZG495_I_Pos.raw	Flublok 2022-2023	ZG495	SF9 recombinant	Injection replicate
Flublok22-23_05_In-Gel_NL_2024-10-25_380-2000_120_HS152535CIT_230min_3750ng_TrypChymo_ZG508_I_Pos.raw	Flublok 2022-2023	ZG508	SF9 recombinant	Injection replicate
Flublok22-23_06_In-Gel_NL_2024-10-25_380-2000_120_HS152535CIT_230min_3750ng_ChymoGluC_ZG509_I_Pos.raw	Flublok 2022-2023	ZG509	SF9 recombinant	Injection replicate
Flublok22-23_07_In-Gel_NL_2024-10-25_380-2000_120_HS152535CIT_230min_3750ng_Chymo_ZG510_I_Pos.raw	Flublok 2022-2023	ZG510	SF9 recombinant	Injection replicate
Flublok22-23_08_In-Gel_NL_2024-10-25_380-2000_120_HS152535CIT_230min_3750ng_alPWT_ZG511_I_Pos.raw	Flublok 2022-2023	ZG511	SF9 recombinant	Injection replicate
Flublok22-23_09_In-Gel_NL_2024-10-25_380-2000_120_HS152535CIT_230min_3750ng_TrypGluC_ZG512_I_Pos.raw	Flublok 2022-2023	ZG512	SF9 recombinant	Digestion replicate
Flublok22-23_10_In-Gel_NL_2024-10-25_380-2000_120_HS152535CIT_230min_3750ng_TrypLysC_ZG513_I_Pos.raw	Flublok 2022-2023	ZG513	SF9 recombinant	Digestion replicate
Flublok22-23_11_In-Gel_NL_2024-10-25_380-2000_120_HS152535CIT_230min_3750ng_TrypChymo_ZG514_I_Pos.raw	Flublok 2022-2023	ZG514	SF9 recombinant	Digestion replicate
Flublok22-23_12_In-Gel_NL_2024-10-25_380-2000_120_HS152535CIT_230min_3750ng_ChymoGluC_ZG515_I_Pos.raw	Flublok 2022-2023	ZG515	SF9 recombinant	Digestion replicate
Flublok22-23_13_In-Gel_NL_2024-10-25_380-2000_120_HS152535CIT_230min_3750ng_Chymo_ZG516_I_Pos.raw	Flublok 2022-2023	ZG516	SF9 recombinant	Digestion replicate
Flublok22-23_14_In-Gel_NL_2024-10-25_380-2000_120_HS152535CIT_230min_3750ng_alPWT_ZG517_I_Pos.raw	Flublok 2022-2023	ZG517	SF9 recombinant	Digestion replicate
Flublok22-23_10_Amicon-Cyr_NL_2023-03-17_380-2000_120_HS152535CIT_230min_2ug_TrypGluC_ZG333_I_Pos.raw	Flublok 2022-2023	ZG333	SF9 recombinant	In-solution digestion
Flublok22-23_06_Amicon-Cyr_NL_2023-03-24_380-2000_120_HS152535CIT_230min_2ug_TrypLysC_ZG334_I_Pos.raw	Flublok 2022-2023	ZG334	SF9 recombinant	In-solution digestion
Flublok22-23_07_Amicon-Cyr_NL_2023-03-24_380-2000_120_HS152535CIT_230min_2ug_TrypChymo_ZG335_I_Pos.raw	Flublok 2022-2023	ZG335	SF9 recombinant	In-solution digestion
Flublok22-23_08_Amicon-Cyr_NL_2023-03-24_380-2000_120_HS152535CIT_230min_2ug_ChymoGluC_ZG336_I_Pos.raw	Flublok 2022-2023	ZG336	SF9 recombinant	In-solution digestion
Flublok22-23_09_Amicon-Cyr_NL_2023-03-24_380-2000_120_HS152535CIT_230min_2ug_Chymo_ZG337_I_Pos.raw	Flublok 2022-2023	ZG337	SF9 recombinant	In-solution digestion
Flublok22-23_10_Amicon-Cyr_NL_2023-03-24_380-2000_120_HS152535CIT_230min_2ug_alPWT_ZG338_I_Pos.raw	Flublok 2022-2023	ZG338	SF9 recombinant	In-solution digestion
Fluad23-24_05_In-Gel_NL_2024-09-18_380-2000_120_HS152535CIT_230min_1250ng_TrypGluC_ZG496_I_Pos.raw	Fluad 2023-2024	ZG496	Native in egg	
Fluad23-24_06_In-Gel_NL_2024-09-18_380-2000_120_HS152535CIT_230min_1250ng_TrypLysC_ZG497_I_Pos.raw	Fluad 2023-2024	ZG497	Native in egg	
Fluad23-24_01_In-Gel_NL_2023-11-27_380-2000_120_HS152535CIT_230min_3750ng_TrypChymo_ZG528_I_Pos.raw	Fluad 2023-2024	ZG528	Native in egg	
Fluad23-24_02_In-Gel_NL_2023-11-27_380-2000_120_HS152535CIT_230min_3750ng_ChymoGluC_ZG529_I_Pos.raw	Fluad 2023-2024	ZG529	Native in egg	
Fluad23-24_03_In-Gel_NL_2023-11-27_380-2000_120_HS152535CIT_230min_3750ng_Chymo_ZG530_I_Pos.raw	Fluad 2023-2024	ZG530	Native in egg	
Fluad23-24_04_In-Gel_NL_2023-11-27_380-2000_120_HS152535CIT_230min_3750ng_alPWT_ZG531_I_Pos.raw	Fluad 2023-2024	ZG531	Native in egg	
Fluad23-24_11_In-Gel_NL_2024-09-18_380-2000_120_HS152535CIT_230min_1250ng_TrypGluC_ZG496_I_Pos.raw	Fluad 2023-2024	ZG496	Native in egg	Injection replicate
Fluad23-24_12_In-Gel_NL_2024-09-18_380-2000_120_HS152535CIT_230min_1250ng_TrypLysC_ZG497_I_Pos.raw	Fluad 2023-2024	ZG497	Native in egg	Injection replicate
Fluad23-24_05_In-Gel_NL_2023-11-27_380-2000_120_HS152535CIT_230min_3750ng_TrypChymo_ZG528_I_Pos.raw	Fluad 2023-2024	ZG528	Native in egg	Injection replicate
Fluad23-24_06_In-Gel_NL_2023-11-27_380-2000_120_HS152535CIT_230min_3750ng_ChymoGluC_ZG529_I_Pos.raw	Fluad 2023-2024	ZG529	Native in egg	Injection replicate
Fluad23-24_07_In-Gel_NL_2023-11-27_380-2000_120_HS152535CIT_230min_3750ng_Chymo_ZG530_I_Pos.raw	Fluad 2023-2024	ZG530	Native in egg	Injection replicate
Fluad23-24_08_In-Gel_NL_2023-11-27_380-2000_120_HS152535CIT_230min_3750ng_alPWT_ZG531_I_Pos.raw	Fluad 2023-2024	ZG531	Native in egg	Injection replicate
NIBSC-Vaccine(A-NewCaledonia-20-1999)_01_Cyr_NL_2023-09-15_380-2000_120_HS152535CIT_230min_3ug_TrypGluC_ZG340_I_Pos.raw	NIBSC (A/NewCaledonia/20/1999)	ZG340	Native in egg	
NIBSC-Vaccine(A-NewCaledonia-20-1999)_02_Cyr_NL_2023-09-15_380-2000_120_HS152535CIT_230min_3ug_TrypLysC_ZG341_I_Pos.raw	NIBSC (A/NewCaledonia/20/1999)	ZG341	Native in egg	
NIBSC-Vaccine(A-NewCaledonia-20-1999)_03_Cyr_NL_2023-09-15_380-2000_120_HS152535CIT_230min_3ug_TrypChymo_ZG342_I_Pos.raw	NIBSC (A/NewCaledonia/20/1999)	ZG342	Native in egg	
NIBSC-Vaccine(A-NewCaledonia-20-1999)_04_Cyr_NL_2023-09-15_380-2000_120_HS152535CIT_230min_3ug_ChymoGluC_ZG343_I_Pos.raw	NIBSC (A/NewCaledonia/20/1999)	ZG343	Native in egg	
NIBSC-Vaccine(A-NewCaledonia-20-1999)_05_Cyr_NL_2023-09-15_380-2000_120_HS152535CIT_230min_3ug_Chymo_ZG344_I_Pos.raw	NIBSC (A/NewCaledonia/20/1999)	ZG344	Native in egg	
NIBSC-Vaccine(A-NewCaledonia-20-1999)_06_Cyr_NL_2023-09-15_380-2000_120_HS152535CIT_230min_3ug_alPWT_ZG345_I_Pos.raw	NIBSC (A/NewCaledonia/20/1999)	ZG345	Native in egg	
NIBSC-Vaccine(A-NewCaledonia-20-1999)_19_Cyr_NL_2023-09-15_380-2000_120_HS152535CIT_230min_3ug_TrypGluC_ZG340-rep2_I_Pos.raw	NIBSC (A/NewCaledonia/20/1999)	ZG340	Native in egg	Injection replicate
NIBSC-Vaccine(A-NewCaledonia-20-1999)_20_Cyr_NL_2023-09-15_380-2000_120_HS152535CIT_230min_3ug_TrypLysC_ZG341-rep2_I_Pos.raw	NIBSC (A/NewCaledonia/20/1999)	ZG341	Native in egg	Injection replicate
NIBSC-Vaccine(A-NewCaledonia-20-1999)_21_Cyr_NL_2023-09-15_380-2000_120_HS152535CIT_230min_3ug_TrypChymo_ZG342-rep2_I_Pos.raw	NIBSC (A/NewCaledonia/20/1999)	ZG342	Native in egg	Injection replicate
NIBSC-Vaccine(A-NewCaledonia-20-1999)_22_Cyr_NL_2023-09-15_380-2000_120_HS152535CIT_230min_3ug_ChymoGluC_ZG343-rep2_I_Pos.raw	NIBSC (A/NewCaledonia/20/1999)	ZG343	Native in egg	Injection replicate
NIBSC-Vaccine(A-NewCaledonia-20-1999)_23_Cyr_NL_2023-09-15_380-2000_120_HS152535CIT_230min_3ug_Chymo_ZG344-rep2_I_Pos.raw	NIBSC (A/NewCaledonia/20/1999)	ZG344	Native in egg	Injection replicate
NIBSC-Vaccine(A-NewCaledonia-20-1999)_24_Cyr_NL_2023-09-15_380-2000_120_HS152535CIT_230min_3ug_alPWT_ZG345-rep2_I_Pos.raw	NIBSC (A/NewCaledonia/20/1999)	ZG345	Native in egg	Injection replicate
NIBSC-Vaccine(A-NewCaledonia-20-1999)_22_Cyr_NL_2022-12-21_380-2000_120_HS152535CIT_230min_3ug_TrypGluC_ZG283_I_Pos.raw	NIBSC (A/NewCaledonia/20/1999)	ZG283	Native in egg	Digestion replicate
NIBSC-Vaccine(A-NewCaledonia-20-1999)_23_Cyr_NL_2022-12-21_380-2000_120_HS152535CIT_230min_3ug_TrypLysC_ZG284_I_Pos.raw	NIBSC (A/NewCaledonia/20/1999)	ZG284	Native in egg	Digestion replicate
NIBSC-Vaccine(A-NewCaledonia-20-1999)_24_Cyr_NL_2022-12-21_380-2000_120_HS152535CIT_230min_3ug_TrypChymo_ZG285_I_Pos.raw	NIBSC (A/NewCaledonia/20/1999)	ZG285	Native in egg	Digestion replicate
NIBSC-Vaccine(A-NewCaledonia-20-1999)_25_Cyr_NL_2022-12-21_380-2000_120_HS152535CIT_230min_3ug_ChymoGluC_ZG286_I_Pos.raw	NIBSC (A/NewCaledonia/20/1999)	ZG286	Native in egg	Digestion replicate
NIBSC-Vaccine(A-NewCaledonia-20-1999)_26_Cyr_NL_2022-12-21_380-2000_120_HS152535CIT_230min_3ug_Chymo_ZG287_I_Pos.raw	NIBSC (A/NewCaledonia/20/1999)	ZG287	Native in egg	Digestion replicate
NIBSC-Vaccine(A-NewCaledonia-20-1999)_27_Cyr_NL_2022-12-21_380-2000_120_HS152535CIT_230min_3ug_alPWT_ZG288_I_Pos.raw	NIBSC (A/NewCaledonia/20/1999)	ZG288	Native in egg	Digestion replicate
NIBSC-Vaccine(A-Philippines-2-1982)_07_Cyr_NL_2023-09-15_380-2000_120_HS152535CIT_230min_3ug_TrypGluC_ZG346_I_Pos.raw	NIBSC (A/Philippines/2/1982)	ZG346	Native in egg	
NIBSC-Vaccine(A-Philippines-2-1982)_08_Cyr_NL_2023-09-15_380-2000_120_HS152535CIT_230min_3ug_TrypLysC_ZG347_I_Pos.raw	NIBSC (A/Philippines/2/1982)	ZG347	Native in egg	
NIBSC-Vaccine(A-Philippines-2-1982)_09_Cyr_NL_2023-09-15_380-2000_120_HS152535CIT_230min_3ug_TrypChymo_ZG348_I_Pos.raw	NIBSC (A/Philippines/2/1982)	ZG348	Native in egg	
NIBSC-Vaccine(A-Philippines-2-1982)_10_Cyr_NL_2023-09-15_380-2000_120_HS152535CIT_230min_3ug_ChymoGluC_ZG349_I_Pos.raw	NIBSC (A/Philippines/2/1982)	ZG349	Native in egg	
NIBSC-Vaccine(A-Philippines-2-1982)_11_Cyr_NL_2023-09-15_380-2000_120_HS152535CIT_230min_3ug_Chymo_ZG350_I_Pos.raw	NIBSC (A/Philippines/2/1982)	ZG350	Native in egg	
NIBSC-Vaccine(A-Philippines-2-1982)_12_Cyr_NL_2023-09-15_380-2000_120_HS152535CIT_230min_3ug_alPWT_ZG351_I_Pos.raw	NIBSC (A/Philippines/2/1982)	ZG351	Native in egg	
NIBSC-Vaccine(A-Philippines-2-1982)_25_Cyr_NL_2023-09-15_380-2000_120_HS152535CIT_230min_3ug_TrypGluC_ZG346-rep2_I_Pos.raw	NIBSC (A/Philippines/2/1982)	ZG346	Native in egg	Injection replicate
NIBSC-Vaccine(A-Philippines-2-1982)_26_Cyr_NL_2023-09-15_380-2000_120_HS152535CIT_230min_3ug_TrypLysC_ZG347-rep2_I_Pos.raw	NIBSC (A/Philippines/2/1982)	ZG347	Native in egg	Injection replicate
NIBSC-Vaccine(A-Philippines-2-1982)_27_Cyr_NL_2023-09-15_380-2000_120_HS152535CIT_230min_3ug_TrypChymo_ZG348-rep2_I_Pos.raw	NIBSC (A/Philippines/2/1982)	ZG348	Native in egg	Injection replicate
NIBSC-Vaccine(A-Philippines-2-1982)_28_Cyr_NL_2023-09-15_380-2000_120_HS152535CIT_230min_3ug_ChymoGluC_ZG349-rep2_I_Pos.raw	NIBSC (A/Philippines/2/1982)	ZG349	Native in egg	Injection replicate
NIBSC-Vaccine(A-Philippines-2-1982)_29_Cyr_NL_2023-09-15_380-2000_120_HS152535CIT_230min_3ug_Chymo_ZG350-rep2_I_Pos.raw	NIBSC (A/Philippines/2/1982)	ZG350	Native in egg	Injection replicate
NIBSC-Vaccine(A-Philippines-2-1982)_30_Cyr_NL_2023-09-15_380-2000_120_HS152535CIT_230min_3ug_alPWT_ZG351-rep2_I_Pos.raw	NIBSC (A/Philippines/2/1982)	ZG351	Native in egg	Injection replicate
NIBSC-Vaccine(A-Philippines-2-1982)_10_Cyr_NL_2022-12-08_380-2000_120_HS152535CIT_230min_3ug_TrypGluC_ZG271_I_Pos.raw	NIBSC (A/Philippines/2/1982)	ZG271	Native in egg	Digestion replicate
NIBSC-Vaccine(A-Philippines-2-1982)_11_Cyr_NL_2022-12-08_380-2000_120_HS152535CIT_230min_3ug_TrypLysC_ZG272_I_Pos.raw	NIBSC (A/Philippines/2/1982)	ZG272	Native in egg	Digestion replicate
NIBSC-Vaccine(A-Philippines-2-1982)_12_Cyr_NL_2022-12-08_380-2000_120_HS152535CIT_230min_3ug_TrypChymo_ZG273_I_Pos.raw	NIBSC (A/Philippines/2/1982)	ZG273	Native in egg	Digestion replicate
NIBSC-Vaccine(A-Philippines-2-1982)_13_Cyr_NL_2022-12-08_380-2000_120_HS152535CIT_230min_3ug_ChymoGluC_ZG274_I_Pos.raw	NIBSC (A/Philippines/2/1982)	ZG274	Native in egg	Digestion replicate
NIBSC-Vaccine(A-Philippines-2-1982)_14_Cyr_NL_2022-12-08_380-2000_120_HS152535CIT_230min_3ug_Chymo_ZG275_I_Pos.raw	NIBSC (A/Philippines/2/1982)	ZG275	Native in egg	Digestion replicate
NIBSC-Vaccine(A-Philippines-2-1982)_15_Cyr_NL_2022-12-08_380-2000_120_HS152535CIT_230min_3ug_alPWT_ZG276_I_Pos.raw	NIBSC (A/Philippines/2/1982)	ZG276	Native in egg	Digestion replicate
NIBSC-Vaccine(A-Switzerland-9715293-2013)_13_Cyr_NL_2023-09-15_380-2000_120_HS152535CIT_230min_3ug_TrypGluC_ZG352_I_Pos.raw	NIBSC (A/Switzerland/9715293/2013)	ZG352	Native in egg	
NIBSC-Vaccine(A-Switzerland-9715293-2013)_14_Cyr_NL_2023-09-15_380-2000_120_HS152535CIT_230min_3ug_TrypLysC_ZG353_I_Pos.raw	NIBSC (A/Switzerland/9715293/2013)	ZG353	Native in egg	
NIBSC-Vaccine(A-Switzerland-9715293-2013)_15_Cyr_NL_2023-09-15_380-2000_120_HS152535CIT_230min_3ug_TrypChymo_ZG354_I_Pos.raw	NIBSC (A/Switzerland/9715293/2013)	ZG354	Native in egg	
NIBSC-Vaccine(A-Switzerland-9715293-2013)_16_Cyr_NL_2023-09-15_380-2000_120_HS152535CIT_230min_3ug_ChymoGluC_ZG355_I_Pos.raw	NIBSC (A/Switzerland/9715293/2013)	ZG355	Native in egg	
NIBSC-Vaccine(A-Switzerland-9715293-2013)_17_Cyr_NL_2023-09-15_380-2000_120_HS152535CIT_230min_3ug_Chymo_ZG356_I_Pos.raw	NIBSC (A/Switzerland/9715293/2013)	ZG356	Native in egg	
NIBSC-Vaccine(A-Switzerland-9715293-2013)_18_Cyr_NL_2023-09-15_380-2000_120_HS152535CIT_230min_3ug_alPWT_ZG357_I_Pos.raw	NIBSC (A/Switzerland/9715293/2013)	ZG357	Native in egg	
NIBSC-Vaccine(A-Switzerland-9715293-2013)_31_Cyr_NL_2023-09-15_380-2000_120_HS152535CIT_230min_3ug_TrypGluC_ZG352-rep2_I_Pos.raw	NIBSC (A/Switzerland/9715293/2013)	ZG352	Native in egg	Injection replicate
NIBSC-Vaccine(A-Switzerland-9715293-2013)_32_Cyr_NL_2023-09-15_380-2000_120_HS152535CIT_230min_3ug_TrypLysC_ZG353-rep2_I_Pos.raw	NIBSC (A/Switzerland/9715293/2013)	ZG353	Native in egg	Injection replicate
NIBSC-Vaccine(A-Switzerland-9715293-2013)_33_Cyr_NL_2023-09-15_380-2000_120_HS152535CIT_230min_3ug_TrypChymo_ZG354-rep2_I_Pos.raw	NIBSC (A/Switzerland/9715293/2013)	ZG354	Native in egg	Injection replicate
NIBSC-Vaccine(A-Switzerland-9715293-2013)_34_Cyr_NL_2023-09-15_380-2000_120_HS152535CIT_230min_3ug_ChymoGluC_ZG355-rep2_I_Pos.raw	NIBSC (A/Switzerland/9715293/2013)	ZG355	Native in egg	Injection replicate
NIBSC-Vaccine(A-Switzerland-9715293-2013)_35_Cyr_NL_2023-09-15_380-2000_120_HS152535CIT_230min_3ug_Chymo_ZG356-rep2_I_Pos.raw	NIBSC (A/Switzerland/9715293/2013)	ZG356	Native in egg	Injection replicate
NIBSC-Vaccine(A-Switzerland-9715293-2013)_36_Cyr_NL_2023-09-15_380-2000_120_HS152535CIT_230min_3ug_alPWT_ZG357-rep2_I_Pos.raw	NIBSC (A/Switzerland/9715293/2013)	ZG357	Native in egg	Injection replicate
NIBSC-Vaccine(A-Switzerland-9715293-2013)_16_Cyr_NL_2022-12-21_380-2000_120_HS152535CIT_230min_3ug_TrypGluC_ZG277_I_Pos.raw	NIBSC (A/Switzerland/9715293/2013)	ZG277	Native in egg	Digestion replicate
NIBSC-Vaccine(A-Switzerland-9715293-2013)_17_Cyr_NL_2022-12-21_380-2000_120_HS152535CIT_230min_3ug_TrypLysC_ZG278_I_Pos.raw	NIBSC (A/Switzerland/9715293/2013)	ZG278	Native in egg	Digestion replicate
NIBSC-Vaccine(A-Switzerland-9715293-2013)_18_Cyr_NL_2022-12-21_380-2000_120_HS152535CIT_230min_3ug_TrypChymo_ZG279_I_Pos.raw	NIBSC (A/Switzerland/9715293/2013)	ZG279	Native in egg	Digestion replicate
NIBSC-Vaccine(A-Switzerland-9715293-2013)_19_Cyr_NL_2022-12-21_380-2000_120_HS152535CIT_230min_3ug_ChymoGluC_ZG280_I_Pos.raw	NIBSC (A/Switzerland/9715293/2013)	ZG280	Native in egg	Digestion replicate
NIBSC-Vaccine(A-Switzerland-9715293-2013)_20_Cyr_NL_2022-12-21_380-2000_120_HS152535CIT_230min_3ug_Chymo_ZG281_I_Pos.raw	NIBSC (A/Switzerland/9715293/2013)	ZG281	Native in egg	Digestion replicate
NIBSC-Vaccine(A-Switzerland-9715293-2013)_21_Cyr_NL_2022-12-21_380-2000_120_HS152535CIT_230min_3ug_alPWT_ZG282_I_Pos.raw	NIBSC (A/Switzerland/9715293/2013)	ZG282	Native in egg	Digestion replicate
CreativeBioVaccine(A-Panama-2007-99)_01_Cyr_NL_2024-05-20_380-2000_120_HS152535CIT_230min_3500ng_TrypGluC_ZG388_I_Pos	Creative Biomart (A/Panama/2007/1999)	ZG388	Native in egg	
CreativeBioVaccine(A-Panama-2007-99)_02_Cyr_NL_2024-05-20_380-2000_120_HS152535CIT_230min_3500ng_TrypLysC_ZG389_I_Pos	Creative Biomart (A/Panama/2007/1999)	ZG389	Native in egg	
CreativeBioVaccine(A-Panama-2007-99)_03_Cyr_NL_2024-05-20_380-2000_120_HS152535CIT_230min_3500ng_TrypChymo_ZG390_I_Pos	Creative Biomart (A/Panama/2007/1999)	ZG390	Native in egg	
CreativeBioVaccine(A-Panama-2007-99)_04_Cyr_NL_2024-05-20_380-2000_120_HS152535CIT_230min_3500ng_ChymoGluC_ZG391_I_Pos	Creative Biomart (A/Panama/2007/1999)	ZG391	Native in egg	
CreativeBioVaccine(A-Panama-2007-99)_05_Cyr_NL_2024-05-20_380-2000_120_HS152535CIT_230min_3500ng_Chymo_ZG392_I_Pos	Creative Biomart (A/Panama/2007/1999)	ZG392	Native in egg	
CreativeBioVaccine(A-Panama-2007-99)_06_Cyr_NL_2024-05-20_380-2000_120_HS152535CIT_230min_3500ng_alPWT_ZG393_I_Pos	Creative Biomart (A/Panama/2007/1999)	ZG393	Native in egg	
CreativeBioVaccine(A-Panama-2007-99)_07_Cyr_NL_2024-05-20_380-2000_120_HS152535CIT_230min_3500ng_TrypGluC_ZG388_I_Pos	Creative Biomart (A/Panama/2007/1999)	ZG388	Native in egg	Injection replicate
CreativeBioVaccine(A-Panama-2007-99)_08_Cyr_NL_2024-05-20_380-2000_120_HS152535CIT_230min_3500ng_TrypLysC_ZG389_I_Pos	Creative Biomart (A/Panama/2007/1999)	ZG389	Native in egg	Injection replicate
CreativeBioVaccine(A-Panama-2007-99)_09_Cyr_NL_2024-05-20_380-2000_120_HS152535CIT_230min_3500ng_TrypChymo_ZG390_I_Pos	Creative Biomart (A/Panama/2007/1999)	ZG390	Native in egg	Injection replicate
CreativeBioVaccine(A-Panama-2007-99)_10_Cyr_NL_2024-05-20_380-2000_120_HS152535CIT_230min_3500ng_ChymoGluC_ZG391_I_Pos	Creative Biomart (A/Panama/2007/1999)	ZG391	Native in egg	Injection replicate
CreativeBioVaccine(A-Panama-2007-99)_11_Cyr_NL_2024-05-20_380-2000_120_HS152535CIT_230min_3500ng_Chymo_ZG392_I_Pos	Creative Biomart (A/Panama/2007/1999)	ZG392	Native in egg	Injection replicate
CreativeBioVaccine(A-Panama-2007-99)_12_Cyr_NL_2024-05-20_380-2000_120_HS152535CIT_230min_3500ng_alPWT_ZG393_I_Pos	Creative Biomart (A/Panama/2007/1999)	ZG393	Native in egg	Injection replicate
CreativeBioVaccine(A-Panama-2007-99)_13_Cyr_NL_2024-05-20_380-2000_120_HS152535CIT_230min_3500ng_TrypGluC_ZG394_I_Pos	Creative Biomart (A/Panama/2007/1999)	ZG394	Native in egg	Digestion replicate
CreativeBioVaccine(A-Panama-2007-99)_14_Cyr_NL_2024-05-20_380-2000_120_HS152535CIT_230min_3500ng_TrypLysC_ZG395_I_Pos	Creative Biomart (A/Panama/2007/1999)	ZG395	Native in egg	Digestion replicate
CreativeBioVaccine(A-Panama-2007-99)_15_Cyr_NL_2024-05-20_380-2000_120_HS152535CIT_230min_3500ng_TrypChymo_ZG396_I_Pos	Creative Biomart (A/Panama/2007/1999)	ZG396	Native in egg	Digestion replicate
CreativeBioVaccine(A-Panama-2007-99)_16_Cyr_NL_2024-05-20_380-2000_120_HS152535CIT_230min_3500ng_ChymoGluC_ZG397_I_Pos	Creative Biomart (A/Panama/2007/1999)	ZG397	Native in egg	Digestion replicate
CreativeBioVaccine(A-Panama-2007-99)_17_Cyr_NL_2024-05-20_380-2000_120_HS152535CIT_230min_3500ng_Chymo_ZG398_I_Pos	Creative Biomart (A/Panama/2007/1999)	ZG398	Native in egg	Digestion replicate
CreativeBioVaccine(A-Panama-2007-99)_18_Cyr_NL_2024-05-20_380-2000_120_HS152535CIT_230min_3500ng_alPWT_ZG399_I_Pos	Creative Biomart (A/Panama/2007/1999)	ZG399	Native in egg	Digestion replicate
CreativeBioVaccine(A-Shandong-9-93)_01_Cyr_NL_2024-06-10_380-2000_120_HS152535CIT_230min_4200ng_TrypGluC_ZG400_I_Pos	Creative Biomart (A/Shandong/9/1993)	ZG400	Native in egg	
CreativeBioVaccine(A-Shandong-9-93)_02_Cyr_NL_2024-06-10_380-2000_120_HS152535CIT_230min_4200ng_TrypLysC_ZG401_I_Pos	Creative Biomart (A/Shandong/9/1993)	ZG401	Native in egg	
CreativeBioVaccine(A-Shandong-9-93)_03_Cyr_NL_2024-06-10_380-2000_120_HS152535CIT_230min_4200ng_TrypChymo_ZG402_I_Pos	Creative Biomart (A/Shandong/9/1993)	ZG402	Native in egg	
CreativeBioVaccine(A-Shandong-9-93)_04_Cyr_NL_2024-06-10_380-2000_120_HS152535CIT_230min_4200ng_ChymoGluC_ZG403_I_Pos	Creative Biomart (A/Shandong/9/1993)	ZG403	Native in egg	
CreativeBioVaccine(A-Shandong-9-93)_05_Cyr_NL_2024-06-10_380-2000_120_HS152535CIT_230min_4200ng_Chymo_ZG404_I_Pos	Creative Biomart (A/Shandong/9/1993)	ZG404	Native in egg	
CreativeBioVaccine(A-Shandong-9-93)_06_Cyr_NL_2024-06-10_380-2000_120_HS152535CIT_230min_4200ng_alPWT_ZG405_I_Pos	Creative Biomart (A/Shandong/9/1993)	ZG405	Native in egg	
CreativeBioVaccine(A-Shandong-9-93)_07_Cyr_NL_2024-06-10_380-2000_120_HS152535CIT_230min_4200ng_TrypGluC_ZG400_I_Pos	Creative Biomart (A/Shandong/9/1993)	ZG400	Native in egg	Injection replicate
CreativeBioVaccine(A-Shandong-9-93)_08_Cyr_NL_2024-06-10_380-2000_120_HS152535CIT_230min_4200ng_TrypLysC_ZG401_I_Pos	Creative Biomart (A/Shandong/9/1993)	ZG401	Native in egg	Injection replicate
CreativeBioVaccine(A-Shandong-9-93)_09_Cyr_NL_2024-06-10_380-2000_120_HS152535CIT_230min_4200ng_TrypChymo_ZG402_I_Pos	Creative Biomart (A/Shandong/9/1993)	ZG402	Native in egg	Injection replicate
CreativeBioVaccine(A-Shandong-9-93)_10_Cyr_NL_2024-06-10_380-2000_120_HS152535CIT_230min_4200ng_ChymoGluC_ZG403_I_Pos	Creative Biomart (A/Shandong/9/1993)	ZG403	Native in egg	Injection replicate
CreativeBioVaccine(A-Shandong-9-93)_11_Cyr_NL_2024-06-10_380-2000_120_HS152535CIT_230min_4200ng_Chymo_ZG404_I_Pos	Creative Biomart (A/Shandong/9/1993)	ZG404	Native in egg	Injection replicate
CreativeBioVaccine(A-Shandong-9-93)_12_Cyr_NL_2024-06-10_380-2000_120_HS152535CIT_230min_4200ng_alPWT_ZG405_I_Pos	Creative Biomart (A/Shandong/9/1993)	ZG405	Native in egg	Injection replicate
CreativeBioVaccine(A-Shandong-9-93)_13_Cyr_NL_2024-06-10_380-2000_120_HS152535CIT_230min_4200ng_TrypGluC_ZG406_I_Pos	Creative Biomart (A/Shandong/9/1993)	ZG406	Native in egg	Digestion replicate
CreativeBioVaccine(A-Shandong-9-93)_14_Cyr_NL_2024-06-10_380-2000_120_HS152535CIT_230min_4200ng_TrypLysC_ZG407_I_Pos	Creative Biomart (A/Shandong/9/1993)	ZG407	Native in egg	Digestion replicate
CreativeBioVaccine(A-Shandong-9-93)_15_Cyr_NL_2024-06-10_380-2000_120_HS152535CIT_230min_4200ng_TrypChymo_ZG408_I_Pos	Creative Biomart (A/Shandong/9/1993)	ZG408	Native in egg	Digestion replicate
CreativeBioVaccine(A-Shandong-9-93)_16_Cyr_NL_2024-06-10_380-2000_120_HS152535CIT_230min_4200ng_ChymoGluC_ZG409_I_Pos	Creative Biomart (A/Shandong/9/1993)	ZG409	Native in egg	Digestion replicate
CreativeBioVaccine(A-Shandong-9-93)_17_Cyr_NL_2024-06-10_380-2000_120_HS152535CIT_230min_4200ng_Chymo_ZG410_I_Pos	Creative Biomart (A/Shandong/9/1993)	ZG410	Native in egg	Digestion replicate
CreativeBioVaccine(A-Shandong-9-93)_18_Cyr_NL_2024-06-10_380-2000_120_HS152535CIT_230min_4200ng_alPWT_ZG411_I_Pos	Creative Biomart (A/Shandong/9/1993)	ZG411	Native in egg	Digestion replicate
CreativeBioVaccine(A-NewCaledonia-20-99)_01_Cyr_NL_2024-06-20_380-2000_120_HS152535CIT_230min_3700ng_TrypGluC_ZG412_I_Pos	Creative Biomart (A/New Caledonia/20/1999)	ZG412	Native in egg	
CreativeBioVaccine(A-NewCaledonia-20-99)_02_Cyr_NL_2024-06-20_380-2000_120_HS152535CIT_230min_3700ng_TrypLysC_ZG413_I_Pos	Creative Biomart (A/New Caledonia/20/1999)	ZG413	Native in egg	
CreativeBioVaccine(A-NewCaledonia-20-99)_03_Cyr_NL_2024-06-20_380-2000_120_HS152535CIT_230min_3700ng_TrypChymo_ZG414_I_Pos	Creative Biomart (A/New Caledonia/20/1999)	ZG414	Native in egg	
CreativeBioVaccine(A-NewCaledonia-20-99)_04_Cyr_NL_2024-06-20_380-2000_120_HS152535CIT_230min_3700ng_ChymoGluC_ZG415_I_Pos	Creative Biomart (A/New Caledonia/20/1999)	ZG415	Native in egg	
CreativeBioVaccine(A-NewCaledonia-20-99)_05_Cyr_NL_2024-06-20_380-2000_120_HS152535CIT_230min_3700ng_Chymo_ZG416_I_Pos	Creative Biomart (A/New Caledonia/20/1999)	ZG416	Native in egg	
CreativeBioVaccine(A-NewCaledonia-20-99)_06_Cyr_NL_2024-06-20_380-2000_120_HS152535CIT_230min_3700ng_alPWT_ZG417_I_Pos	Creative Biomart (A/New Caledonia/20/1999)	ZG417	Native in egg	
CreativeBioVaccine(A-NewCaledonia-20-99)_07_Cyr_NL_2024-06-20_380-2000_120_HS152535CIT_230min_3700ng_TrypGluC_ZG412_I_Pos	Creative Biomart (A/New Caledonia/20/1999)	ZG412	Native in egg	Injection replicate
CreativeBioVaccine(A-NewCaledonia-20-99)_08_Cyr_NL_2024-06-20_380-2000_120_HS152535CIT_230min_3700ng_TrypLysC_ZG413_I_Pos	Creative Biomart (A/New Caledonia/20/1999)	ZG413	Native in egg	Injection replicate
CreativeBioVaccine(A-NewCaledonia-20-99)_09_Cyr_NL_2024-06-20_380-2000_120_HS152535CIT_230min_3700ng_TrypChymo_ZG414_I_Pos	Creative Biomart (A/New Caledonia/20/1999)	ZG414	Native in egg	Injection replicate
CreativeBioVaccine(A-NewCaledonia-20-99)_10_Cyr_NL_2024-06-20_380-2000_120_HS152535CIT_230min_3700ng_ChymoGluC_ZG415_I_Pos	Creative Biomart (A/New Caledonia/20/1999)	ZG415	Native in egg	Injection replicate
CreativeBioVaccine(A-NewCaledonia-20-99)_11_Cyr_NL_2024-06-20_380-2000_120_HS152535CIT_230min_3700ng_Chymo_ZG416_I_Pos	Creative Biomart (A/New Caledonia/20/1999)	ZG416	Native in egg	Injection replicate
CreativeBioVaccine(A-NewCaledonia-20-99)_12_Cyr_NL_2024-06-20_380-2000_120_HS152535CIT_230min_3700ng_alPWT_ZG417_I_Pos	Creative Biomart (A/New Caledonia/20/1999)	ZG417	Native in egg	Injection replicate
CreativeBioVaccine(A-NewCaledonia-20-99)_13_Cyr_NL_2024-06-20_380-2000_120_HS152535CIT_230min_3700ng_TrypGluC_ZG418_I_Pos	Creative Biomart (A/New Caledonia/20/1999)	ZG418	Native in egg	Digestion replicate
CreativeBioVaccine(A-NewCaledonia-20-99)_14_Cyr_NL_2024-06-20_380-2000_120_HS152535CIT_230min_3700ng_TrypLysC_ZG419_I_Pos	Creative Biomart (A/New Caledonia/20/1999)	ZG419	Native in egg	Digestion replicate
CreativeBioVaccine(A-NewCaledonia-20-99)_15_Cyr_NL_2024-06-20_380-2000_120_HS152535CIT_230min_3700ng_TrypChymo_ZG420_I_Pos	Creative Biomart (A/New Caledonia/20/1999)	ZG420	Native in egg	Digestion replicate
CreativeBioVaccine(A-NewCaledonia-20-99)_16_Cyr_NL_2024-06-20_380-2000_120_HS152535CIT_230min_3700ng_ChymoGluC_ZG421_I_Pos	Creative Biomart (A/New Caledonia/20/1999)	ZG421	Native in egg	Digestion replicate
CreativeBioVaccine(A-NewCaledonia-20-99)_17_Cyr_NL_2024-06-20_380-2000_120_HS152535CIT_230min_3700ng_Chymo_ZG422_I_Pos	Creative Biomart (A/New Caledonia/20/1999)	ZG422	Native in egg	Digestion replicate
CreativeBioVaccine(A-NewCaledonia-20-99)_18_Cyr_NL_2024-06-20_380-2000_120_HS152535CIT_230min_3700ng_alPWT_ZG423_I_Pos	Creative Biomart (A/New Caledonia/20/1999)	ZG423	Native in egg	Digestion replicate
Novavax-Covid19-23-24_01_In-Gel_NL_2024-12-06_380-2000_120_HS152535CIT_230min_1875ng_TrypGluC_ZG532_I_Pos	Novavax Covid-19 2023-2024	ZG532	SF9 recombinant	
Novavax-Covid19-23-24_02_In-Gel_NL_2024-12-06_380-2000_120_HS152535CIT_230min_1875ng_TrypLysC_ZG533_I_Pos	Novavax Covid-19 2023-2024	ZG533	SF9 recombinant	
Novavax-Covid19-23-24_03_In-Gel_NL_2024-12-06_380-2000_120_HS152535CIT_230min_1875ng_TrypChymo_ZG534_I_Pos	Novavax Covid-19 2023-2024	ZG534	SF9 recombinant	
Novavax-Covid19-23-24_04_In-Gel_NL_2024-12-06_380-2000_120_HS152535CIT_230min_1875ng_ChymoGluC_ZG535_I_Pos	Novavax Covid-19 2023-2024	ZG535	SF9 recombinant	
Novavax-Covid19-23-24_05_In-Gel_NL_2024-12-06_380-2000_120_HS152535CIT_230min_1875ng_Chymo_ZG536_I_Pos	Novavax Covid-19 2023-2024	ZG536	SF9 recombinant	
Novavax-Covid19-23-24_01_In-Gel_NL_2024-12-23_380-2000_120_HS152535CIT_230min_1875ng_alPWT_ZG537_I_Pos	Novavax Covid-19 2023-2024	ZG537	SF9 recombinant	
Novavax-Covid19-23-24_07_In-Gel_NL_2024-12-06_380-2000_120_HS152535CIT_230min_1875ng_TrypGluC_ZG532_I_Pos	Novavax Covid-19 2023-2024	ZG532	SF9 recombinant	Injection replicate
Novavax-Covid19-23-24_08_In-Gel_NL_2024-12-06_380-2000_120_HS152535CIT_230min_1875ng_TrypLysC_ZG533_I_Pos	Novavax Covid-19 2023-2024	ZG533	SF9 recombinant	Injection replicate
Novavax-Covid19-23-24_09_In-Gel_NL_2024-12-06_380-2000_120_HS152535CIT_230min_1875ng_TrypChymo_ZG534_I_Pos	Novavax Covid-19 2023-2024	ZG534	SF9 recombinant	Injection replicate
Novavax-Covid19-23-24_10_In-Gel_NL_2024-12-06_380-2000_120_HS152535CIT_230min_1875ng_ChymoGluC_ZG535_I_Pos	Novavax Covid-19 2023-2024	ZG535	SF9 recombinant	Injection replicate
Novavax-Covid19-23-24_11_In-Gel_NL_2024-12-06_380-2000_120_HS152535CIT_230min_1875ng_Chymo_ZG536_I_Pos	Novavax Covid-19 2023-2024	ZG536	SF9 recombinant	Injection replicate
Novavax-Covid19-23-24_02_In-Gel_NL_2024-12-23_380-2000_120_HS152535CIT_230min_1875ng_alPWT_ZG537_I_Pos	Novavax Covid-19 2023-2024	ZG537	SF9 recombinant	Injection replicate
Shingrix_03_Cyr_NL_2024-12-23_380-2000_120_HS152535CIT_230min_2ug_TrypGluC_ZG538_I_Pos	Shingrix	ZG538	CHO recombinant	
Shingrix_04_Cyr_NL_2024-12-23_380-2000_120_HS152535CIT_230min_2ug_TrypLysC_ZG539_I_Pos	Shingrix	ZG539	CHO recombinant	
Shingrix_05_Cyr_NL_2024-12-23_380-2000_120_HS152535CIT_230min_2ug_TrypChymo_ZG540_I_Pos	Shingrix	ZG540	CHO recombinant	
Shingrix_06_Cyr_NL_2024-12-23_380-2000_120_HS152535CIT_230min_2ug_ChymoGluC_ZG541_I_Pos	Shingrix	ZG541	CHO recombinant	
Shingrix_07_Cyr_NL_2024-12-23_380-2000_120_HS152535CIT_230min_2ug_Chymo_ZG542_I_Pos	Shingrix	ZG542	CHO recombinant	
Shingrix_08_Cyr_NL_2024-12-23_380-2000_120_HS152535CIT_230min_2ug_alPWT_ZG543_I_Pos	Shingrix	ZG543	CHO recombinant	
Shingrix_09_Cyr_NL_2024-12-23_380-2000_120_HS152535CIT_230min_2ug_TrypGluC_ZG538_I_Pos	Shingrix	ZG538	CHO recombinant	Injection replicate
Shingrix_10_Cyr_NL_2024-12-23_380-2000_120_HS152535CIT_230min_2ug_TrypLysC_ZG539_I_Pos	Shingrix	ZG539	CHO recombinant	Injection replicate
Shingrix_11_Cyr_NL_2024-12-23_380-2000_120_HS152535CIT_230min_2ug_TrypChymo_ZG540_I_Pos	Shingrix	ZG540	CHO recombinant	Injection replicate
Shingrix_12_Cyr_NL_2024-12-23_380-2000_120_HS152535CIT_230min_2ug_ChymoGluC_ZG541_I_Pos	Shingrix	ZG541	CHO recombinant	Injection replicate
Shingrix_13_Cyr_NL_2024-12-23_380-2000_120_HS152535CIT_230min_2ug_Chymo_ZG542_I_Pos	Shingrix	ZG542	CHO recombinant	Injection replicate
Shingrix_14_Cyr_NL_2024-12-23_380-2000_120_HS152535CIT_230min_2ug_alPWT_ZG543_I_Pos	Shingrix	ZG543	CHO recombinant	Injection replicate
Shingrix_15_Cyr_NL_2024-12-23_380-2000_120_HS152535CIT_230min_2ug_TrypGluC_ZG544_I_Pos	Shingrix	ZG544	CHO recombinant	Digestion replicate
Shingrix_16_Cyr_NL_2024-12-23_380-2000_120_HS152535CIT_230min_2ug_TrypLysC_ZG545_I_Pos	Shingrix	ZG545	CHO recombinant	Digestion replicate
Shingrix_17_Cyr_NL_2024-12-23_380-2000_120_HS152535CIT_230min_2ug_TrypChymo_ZG546_I_Pos	Shingrix	ZG546	CHO recombinant	Digestion replicate
Shingrix_18_Cyr_NL_2024-12-23_380-2000_120_HS152535CIT_230min_2ug_ChymoGluC_ZG547_I_Pos	Shingrix	ZG547	CHO recombinant	Digestion replicate
Shingrix_19_Cyr_NL_2024-12-23_380-2000_120_HS152535CIT_230min_2ug_Chymo_ZG548_I_Pos	Shingrix	ZG548	CHO recombinant	Digestion replicate
Shingrix_20_Cyr_NL_2024-12-23_380-2000_120_HS152535CIT_230min_2ug_alPWT_ZG549_I_Pos	Shingrix	ZG549	CHO recombinant	Digestion replicate

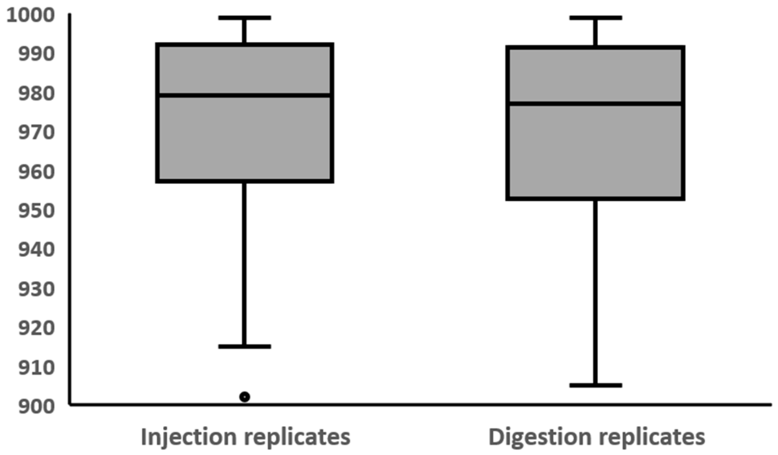

Figure S5. **Box and Whisker Plot of Replicate Injections and Replicate Digestions**
This figure demonstrates the distribution of similarity scores when comparing equivalent GADS between replicate injections and replicate digestions. The median similarity score for injection replicates is 979 and for digestion replicates is 977.

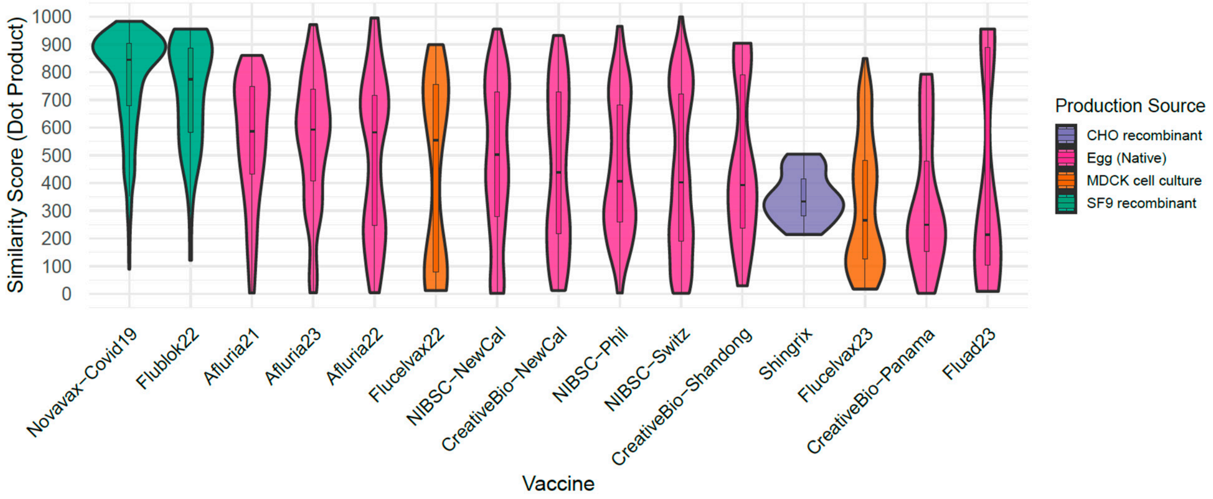

Figure S6. **Intra-protein variation of glycosylation distribution for each vaccine**.
This violin plot summarizes the intra-protein factor of Figure 3. Comparisons are limited to the same protein and strain for each vaccine. Purple represents antigens expressed from a CHO cell recombinant system, pink represents antigens propagated natively in embryonated hen eggs, orange represents antigens propagated natively in MDCK cell culture, and green represents antigens that are expressed from an Sf9 cell recombinant system. Numbers after vaccine names represent the year the vaccine was produced. Box-and-whisker markers plots are included within each violin to show quartiles of the distribution.

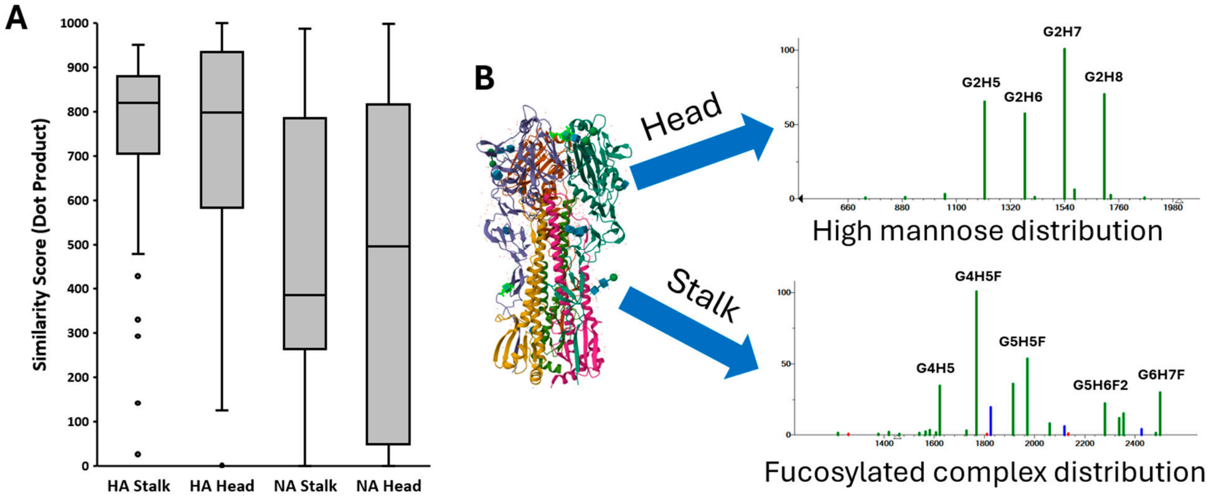

Figure S7. **Correlation between glycosylation distribution similarity and site position from different sites on the same protein**.

Note that this data corresponds to the intra-protein similarity scores reported in Figure 3. A) Intra-protein glycosylation similarity was compared for the head and stalk regions of hemagglutinin and neuraminidase. B) Glycosylation distributions are commonly high mannose type distributions in the head region and of fucosylated complex distributions in the stalk region of hemagglutinin.

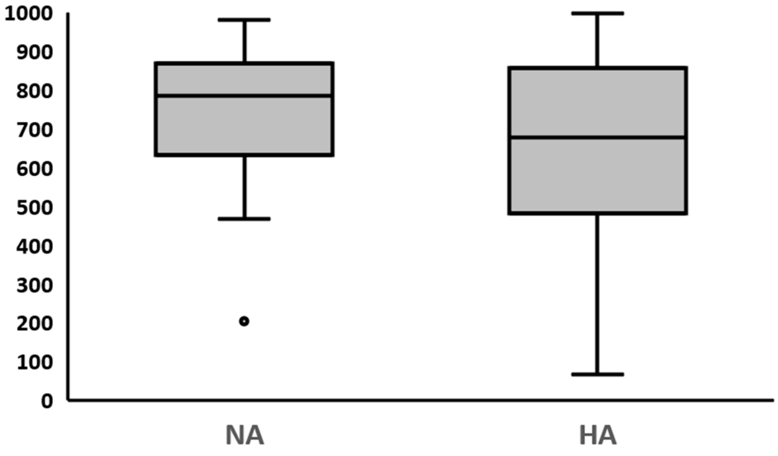

Figure S8. **Box and Whisker Plot of Intra-protein Variation within Neuraminidase and Hemagglutinin**
This figure demonstrates the distribution of similarity scores when comparing glycosylation sites across various sites on the same protein and within the same strain of influenza for only egg-based quadrivalent vaccines. The median similarity score for neuraminidase is 770 and for hemagglutinin is 678.

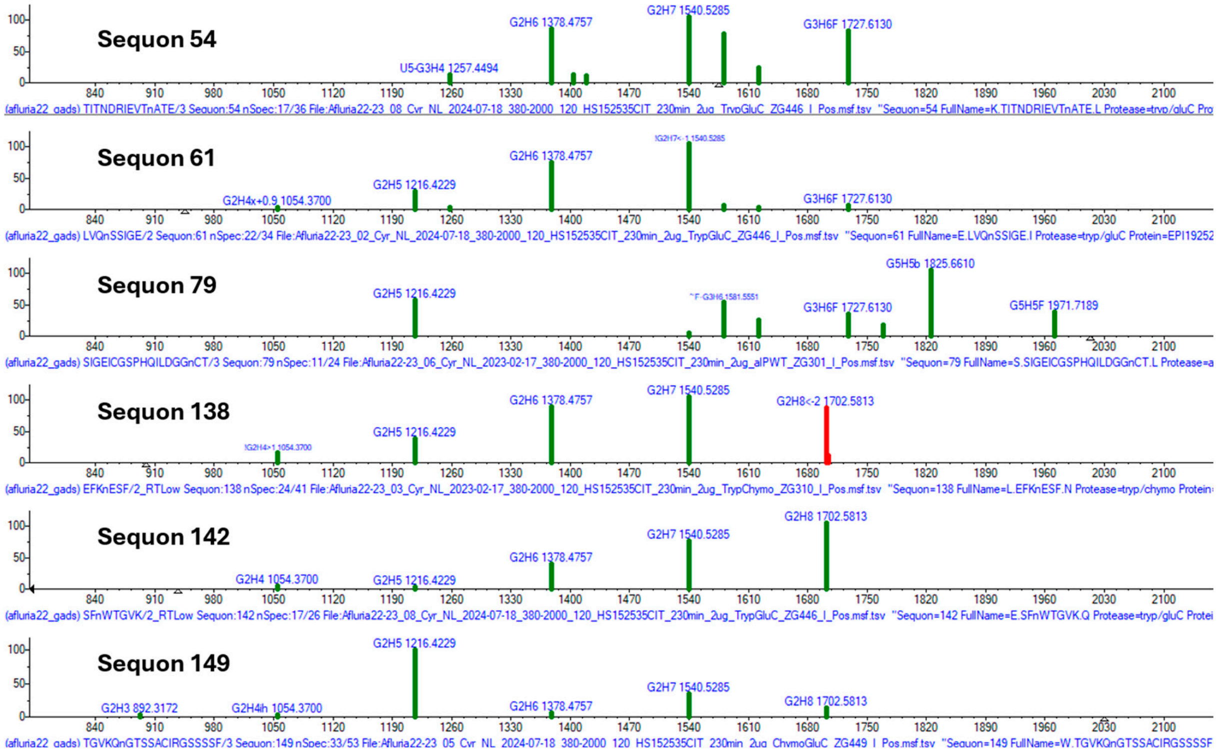

Figure S9. **GADS Comparison of the HA Head Region in Afluria 2022-2023 Formulation**
This is a depiction of glycosylation distributions for glycosylation sites within the HA head region of Afluria 2022-2023 formulation strain A/Darwin/6/2021. Note the primary glycans associated with these glycosylation sites are high-mannose with some monofucosylated complex glycans.

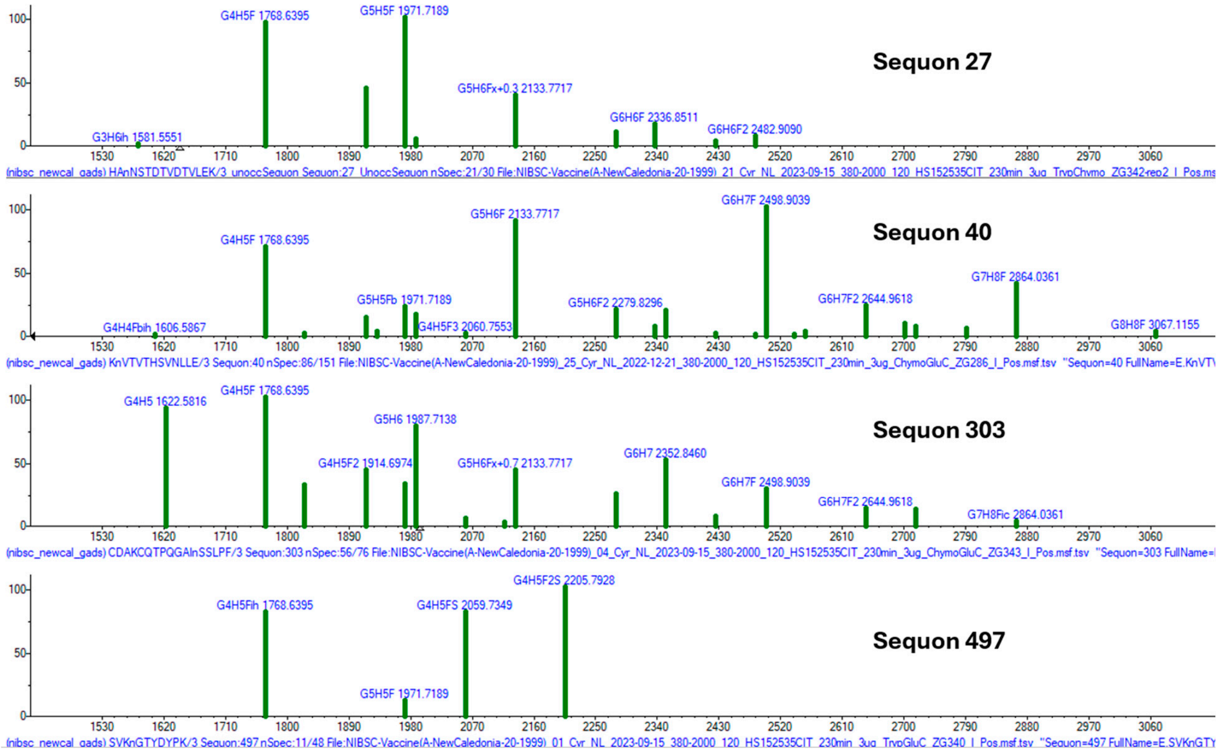

Figure S10. **GADS Comparison of the HA Stalk Region in A/NewCaledonia/20/1999**
This is a depiction of glycosylation distributions for glycosylation sites within the HA stalk region of A/NewCaledonia/20/1999 produced by NIBSC. Note the primary glycans associated with these glycosylation sites are monofucosylated complex glycans with four to six HexNAc residues.

Vaccine	Strains	Glycosylation sites
Afluria Quadrivalent 2021	A/Victoria/2570/2019	27 28 40 104 179 293 304 498 557
	A/Cambodia/e0826360/2020	24 38 54 61 79 138 142 149 174 181 262 301 499
	B/Victoria/705/2018	40 74 160 178 245 316 345 504 530 543
	B/Phuket/3073/2013	40 74 160 182 211 318 347 506 532 545 577
Afluria Quadrivalent 2022	A/Victoria/2570/2019	27 28 40 104 179 293 304 498 557
	A/Darwin/6/2021	24 38 54 61 79 138 142 149 181 262 301 499
	B/Austria/1359417/2021	40 74 160 178 245 316 345 504 530 543 575
	B/Phuket/3073/2013	40 74 160 182 211 318 347 506 532 545 577
Afluria Quadrivalent 2023	A/Victoria/4897/2022	27 28 40 104 179 293 304 498 557
	A/Darwin/6/2021	24 38 54 61 79 138 142 149 181 262 301 499
	B/Austria/1359417/2021	40 74 160 178 245 316 345 504 530 543 575
	B/Phuket/3073/2013	40 74 160 182 211 318 347 506 532 545 577
Flublok Quadrivalent 2022	A/Wisconsin/588/2019	27 28 40 104 179 293 304 498 557
	A/Darwin/6/2021	24 38 54 61 79 138 142 149 181 262 301 499
	B/Austria/1359417/2021	40 74 160 178 245 316 345 504 530 543 575
	B/Phuket/3073/2013	40 74 160 182 211 318 347 506 532 545 577
Flucelvax Quadrivalent 2022	A/Delaware/55/2019	27 28 40 104 179 293 304 498 557
	A/Darwin/11/2021	24 38 54 61 79 138 142 149 181 301 499
	B/Singapore/WUH4618/2021	40 74 160 178 245 316 345 504 530 543 575
	B/Singapore/INFTT-16-0610/2016	40 74 160 182 318 347 506 532 545 577
Flucelvax Quadrivalent 2023	A/Georgia/12/2022	27 28 40 104 179 293 304 498 557
	A/Darwin/11/2021	24 38 54 61 79 138 142 149 181 301 499
	B/Singapore/WUH4618/2021	40 74 160 178 245 316 345 504 530 543 575
	B/Singapore/INFTT-16-0610/2016	40 74 160 182 318 347 506 532 545 577
Fluad Quadrivalent 2023	A/Victoria/4897/2022	27 28 40 104 179 293 304 498 557
	A/Darwin/6/2021	24 38 54 61 79 138 142 149 181 262 301 499
	B/Austria/1359417/2021	40 74 160 178 245 316 345 504 530 543 575
	B/Phuket/3073/2013	40 74 160 182 211 318 347 506 532 545 577

Figure S11. **Glycosylation site alignment for hemagglutinin among quadrivalent influenza vaccines** Protein sequences from all strains of seven influenza vaccines from hemagglutinin were processed by the Clustal Omega program to assess position and overlap. Numbers represent amino acid position of glycosylation sites and boxes indicate overlapping (sequence aligned) glycosylation sites. Red boxes indicate glycosylation sites that are indistinguishable between strains due to identical peptide sequences. Green boxes indicate glycosylation sites that are overlapping in sequence, and therefore considered homologous, but are distinguishable in sequence.

Vaccine	Strains	Glycosylation sites
Afluria Quadrivalent 2021	A/Victoria/2570/2019	42 50 58 63 68 88 146 235
	A/Cambodia/e0826360/2020	61 70 86 146 200 234 245 367 463
	B/Victoria/705/2018	56 64 144 284
	B/Phuket/3073/2013	56 64 144 284 463
Afluria Quadrivalent 2022	A/Victoria/2570/2019	42 50 58 63 68 88 146 235
	A/Darwin/6/2021	61 70 86 146 200 234 245 367 463
	B/Austria/1359417/2021	56 64 144 284
	B/Phuket/3073/2013	56 64 144 284 463
Afluria Quadrivalent 2023	A/Victoria/4897/2022	42 58 63 68 88 146 235
	A/Darwin/6/2021	61 70 86 146 200 234 245 367 463
	B/Austria/1359417/2021	56 64 144 284
	B/Phuket/3073/2013	56 64 144 284 463
Flublok Quadrivalent 2022	A/Wisconsin/588/2019	42 50 58 63 68 88 146 235
	A/Darwin/6/2021	61 70 86 146 200 234 245 367 463
	B/Austria/1359417/2021	56 64 144 284
	B/Phuket/3073/2013	56 64 144 284 463
Flucelvax Quadrivalent 2022	A/Delaware/55/2019	42 50 58 63 68 88 146 235
	A/Darwin/11/2021	61 70 86 146 200 234 245 367 463
	B/Singapore/WUH4618/2021	56 64 145 285
	B/Singapore/INFTT-16-0610/2016	56 64 144 284 463
Flucelvax Quadrivalent 2023	A/Georgia/12/2022	42 50 58 63 68 88 146 235
	A/Darwin/11/2021	61 70 86 146 200 234 245 367 463
	B/Singapore/WUH4618/2021	56 64 145 285
	B/Singapore/INFTT-16-0610/2016	56 64 144 284 463
Fluad Quadrivalent 2023	A/Victoria/4897/2022	42 58 63 68 88 146 235
	A/Darwin/6/2021	61 70 86 146 200 234 245 367 463
	B/Austria/1359417/2021	56 64 144 284
	B/Phuket/3073/2013	56 64 144 284 463

Figure S12. **Glycosylation site alignment for neuraminidase among quadrivalent influenza vaccines** Protein sequences from all strains of seven influenza vaccines from neuraminidase were processed by the Clustal Omega program to assess position and overlap. See figure S11 for details of figure.

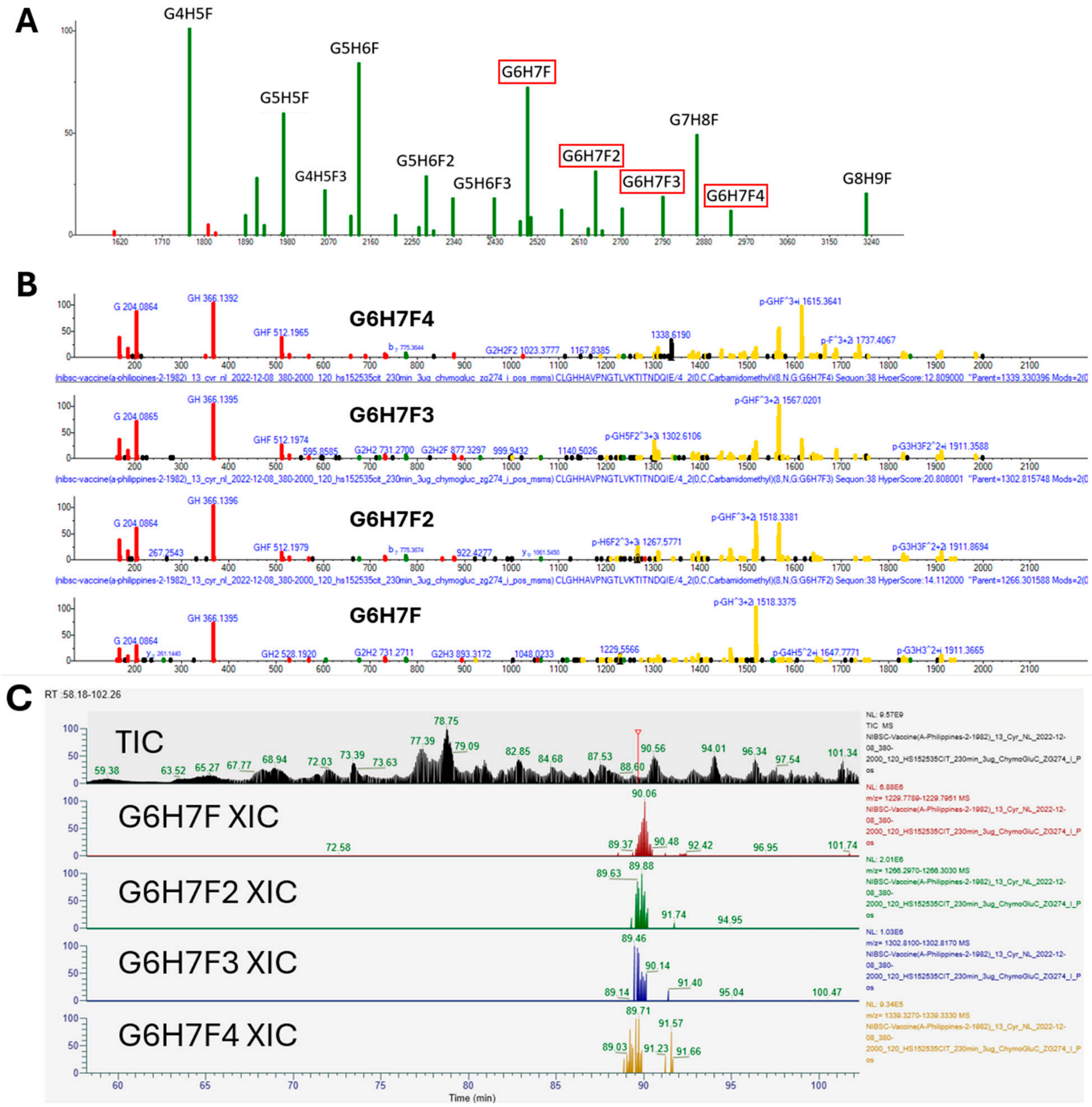

Figure S13. **Illustration of verification of multiply-fucosylated glycopeptide spectra**
Glycopeptides that were identified with multiple fucose residues were verified to prove the absence of sialic acid residues using oxonium ions, Y ions, and retention time. A) GADS of HA in NIBSC vaccine, strain A/Philippines/2/1982, at position 38. Glycans highlighted in red are in question in panels B and C. B) Four MS^2 spectra representing the four glycans in panel A. Note the lack of sialyl oxonium ions at 274 and 292 m/z and the increasing abundance of GHF oxonium ion at 512 m/z with increasing fucosylation. Also note the abundant Y ions denoting p-GHF. C) A total ion chromatogram and four extracted ion chromatograms representing the four glycans highlighted in panel A. Note the lack of change in retention time for these glycopeptides, indicating lack of sialic acid additions.

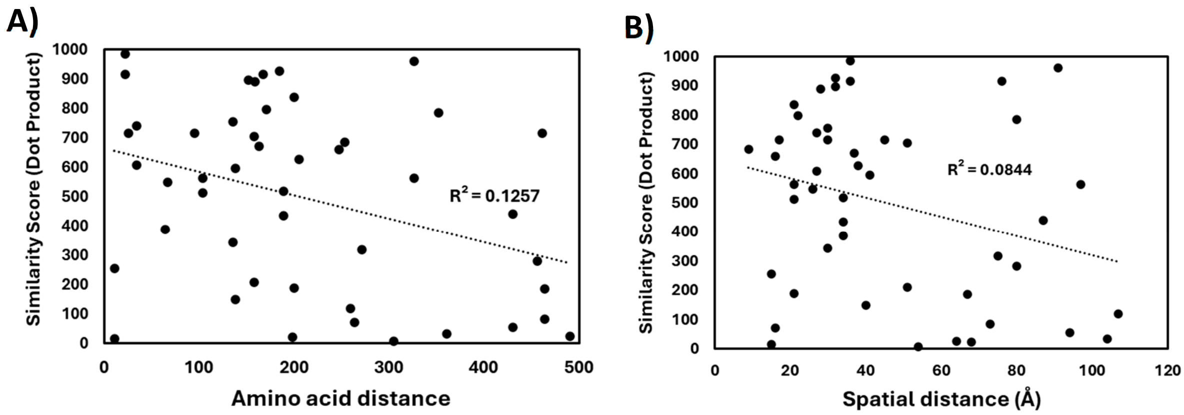

Figure S14. **GADS similarity as a function of glycosylation site distance**
Glycosylation patterns were compared for different sites on the same protein and plotted by amino acid distance (A) and spatial distance (B). While the overall trend shows a decline in similarity with larger distances, the correlation is not strong ($R^2 = 0.1257$ and 0.0844).

Table S3. Summary of all glycosylation comparisons made in this study at the protein level

Factor	Sub-category	Vaccines included	Virus components included	Proteins included
Replicates	Injection Replicates	AFLQ-21	All four	HA
				NA
		AFLQ-22	All four	HA
				NA
		AFLQ-23	All four	HA
				NA
		FBLK-22	All four	HA
				NA
		FCVX-22	All four	HA
				NA
		FCVX-23	All four	HA
				NA
		FLAD-23	All four	HA
				NA
		NIBSC-NC99	A/NewCaledonia/20/1999	HA
				NA
		NIBSC-PH82	A/Philippines/2/1982	HA
				NA
		NIBSC-SW13	A/Switzerland/9715293/2013	HA
				NA
		CB-PA99	A/Panama/2007/1999	HA
				NA
		CB-NC99	A/NewCaledonia/20/1999	HA
				NA
		CB-SD93	A/Shandong/9/1993	HA
				NA
		NVX-23	Omicron XBB.1.5	S
		SHGX	N/A	gE
	Digestion Replicates	AFLQ-21	All four	HA
				NA
		AFLQ-22	All four	HA
				NA
		AFLQ-23	All four	HA
				NA
		FBLK-22	All four	HA
				NA
		FCVX-22	All four	HA
				NA
		FCVX-23	All four	HA
				NA
		NIBSC-NC99	A/NewCaledonia/20/1999	HA
				NA
		NIBSC-PH82	A/Philippines/2/1982	HA
				NA
		NIBSC-SW13	A/Switzerland/9715293/2013	HA
				NA
		CB-PA99	A/Panama/2007/1999	HA
				NA
		CB-NC99	A/NewCaledonia/20/1999	HA
				NA
		CB-SD93	A/Shandong/9/1993	HA
				NA
		SHGX	N/A	gE
Year-to-year	N/A	AFLQ-21; AFLQ-22	A/Victoria/2570/2019	HA
				NA
			B/Phuket/3073/2013	HA
				NA
		AFLQ-22; AFLQ-23	A/Darwin/6/2021	HA
				NA
			B/Austria/1359417/2021	HA
				NA
			B/Phuket/3073/2013	HA
				NA
		FCVX-22; FCVX-23	A/Darwin/11/2021	HA
				NA
			B/Singapore/WUH4618/2021	HA
				NA
			B/Singapore/INFTT-16-0610/2016	HA
				NA
Supplier	N/A	AFLQ-23; FLAD-23	A/Victoria/4897/2022	HA
				NA
		AFLQ-22 and AFLQ-23; FLAD-23	A/Darwin/6/2021	HA
				NA
		AFLQ-22 and AFLQ-23; FLAD-23	B/Austria/1359417/2021	HA
				NA
		AFLQ-21 andAFLQ-22 and AFLQ-23; FLAD-23	B/Phuket/3073/2013	HA
				NA
		NIBSC-NC99; CB-NC99	A/NewCaledonia/20/1999	HA
				NA
Intra-protein	N/A	AFLQ-21	All four	HA
				NA
		AFLQ-22	All four	HA
				NA
		AFLQ-23	All four	HA
				NA
		FBLK-22	All four	HA
				NA
		FCVX-22	All four	HA
				NA
		FCVX-23	All four	HA
				NA
		FLAD-23	All four	HA
				NA
		NIBSC-NC99	A/NewCaledonia/20/1999	HA
				NA
		NIBSC-PH82	A/Philippines/2/1982	HA
				NA
		NIBSC-SW13	A/Switzerland/9715293/2013	HA
				NA
		CB-PA99	A/Panama/2007/1999	HA
				NA
		CB-NC99	A/NewCaledonia/20/1999	HA
				NA
		CB-SD93	A/Shandong/9/1993	HA
				NA
		NVX-23	Omicron XBB.1.5	S
		SHGX	N/A	gE
Inter-strain	Homologous sites	AFLQ-21	All four	HA
				NA
		AFLQ-22	All four	HA
				NA
		AFLQ-23	All four	HA
				NA
		FBLK-22	All four	HA
				NA
		FCVX-22	All four	HA
				NA
		FCVX-23	All four	HA
				NA
		FLAD-23	All four	HA
				NA
	Non-homologous sites	AFLQ-21	All four	HA
				NA
		AFLQ-22	All four	HA
				NA
		AFLQ-23	All four	HA
				NA
		FBLK-22	All four	HA
				NA
		FCVX-22	All four	HA
				NA
		FCVX-23	All four	HA
				NA
		FLAD-23	All four	HA
				NA
Source	Egg vs MDCK	Egg-based quadrivalent vaccines; FCVX-22 and FCVX-23	No identical strains were present between these vaccines. Therefore, homologous sites with identical peptide sequences were compared between equivelant subtypes.	HA
				NA
	Egg vs Sf9	Egg-based quadrivalent vaccines; FBLK-22	Few identical strains were present between these vaccines. Therefore, homologous sites with identical peptide sequences were compared between equivelant subtypes.	HA
				NA
	MDCK vs Sf9	FCVX-22 and FCVX-23; FBLK-22	No identical strains were present between these vaccines. Therefore, homologous sites with identical peptide sequences were compared between equivelant subtypes.	HA
				NA
